# Utilization of road dust chemical profiles for source identification and human health impact assessment

**DOI:** 10.1038/s41598-020-71180-x

**Published:** 2020-08-31

**Authors:** Eun-Ah Kim, Byumseok Koh

**Affiliations:** 1 Chemical Safety Research Center, Korea Research Institute of Chemical Technology, 141 Gajeong-ro, Yuseong-gu, Daejeon, 34114 Republic of Korea; 2 National Assembly Futures Institute, Members Office Bldg, 1 Uisadang-daero, Yeongdeungpo-gu, Seoul, 07233 Republic of Korea; 3 Bio Platform Technology Research Center, Korea Research Institute of Chemical Technology, 141 Gajeong-ro, Yuseong-gu, Daejeon, 34114 Republic of Korea

**Keywords:** Environmental sciences, Natural hazards

## Abstract

This study investigated the chemical profiles of fine urban road dust as a set of indicators for major air pollutants at sampling sites or as proxies for potential human health impacts. We examined the chemical compositions of fine particles (< 100 μm) or re-suspended ultrafine particles (< 2.5 μm) in the urban road dust collected from the cities with major emission sources of CO, NH_3_, NO_x_, PM_2.5_, SO_x_, and volatile organic compounds. The elemental compositions, including metal contents and volatile or semi-volatile organic compound species were determined to constitute comprehensive chemical profiles of the solid road dust samples. The water-extractable organic compounds and fluorescent species of the size-fractionated re-suspended fine particulate matter (RPM) were also incorporated in the chemical profiles. The metal content and aliphatic hydrocarbons could partly distinguish emission sources, and clearer distinctions were achieved with the inclusion of fluorescence excitation-emission matrix (EEM) results. The dose–response test results showed positive correlations between cytotoxicity and relative abundance of hydrocarbons or metal contents of urban road dust. The set of chemical profiles suggested in this study could be further utilized for site identification or human health impact assessment using urban road dust.

## Introduction

Chemical profiles of urban road dust can be utilized not only for assessing health risks but also for identifying the sources of the dust which accumulates chemical ingredients originated from the local environment in close vicinity. The air pollutants may influence the chemical compositions of dust in multiple ways. Both adsorption of air pollutant to fine dust particles and absorption of ambient particulate matter such as PM_10_ to road dust could contribute to partitioning^1^ of air pollutants to road dust. Deposition of ambient particulate matter on road dust could be another pathway that air pollutants influence the chemical compositions of road dust. Wang et al.^2^ investigated factors associated with adsorption of polycyclic aromatic hydrocarbons on road dust. Absorption or deposition of aggregated PM_10_ on road dust may also add air pollutants to road dust particles because a great portion of PM_10_ results from main gaseous precursors^3,4^, such as CO, SO_x_, NO_x_, NH_3_, and VOCs in the atmosphere. These series of findings support the hypothesis of this study that fine road dust can store information about the major emission sources in vicinity.

The fact that chemical composition of road dust is related to a footprint of the local chemical environment forms the basis of environmental soil forensics^5^. The large surface areas of the fine particle fractions in urban road dust could store rich information about the surrounding chemical environment. The elemental composition^6–8^ of urban road dust contains information about its origin, such as the earth’s crust (Mg, Al, Si, Ca, and Fe)^9^. Trace elemental analysis of fine road dust particles could reveal anthropogenic sources, such as traffic emissions (Cu, Mn, Zn, and Pb)^10^, including car exhaust gas and particles from tire or brake abrasion, and other industrial emissions (Cr, Ni, Sr, and Zr). Therefore, the elemental composition of road dust or soil could provide information documenting the characteristics of the local chemical environment.

Parallel factor (PARAFAC) analysis based on fluorescence excitation-emission matrices (EEMs) has been shown to be useful for the pseudo-quantitative source apportionment of dissolved organic matter^11^ of terrestrial and microbial origin and from anthropogenic inputs^12,13^. PARAFAC analysis produces several principal components, and their linear combinations can explain an entire EEM set with negligible residuals of errors. Here, we extended the applicability of PARAFAC by using fluorescence EEM spectroscopy to classify the origins of the water-extractable organic matter contained in re-suspended urban road dust.

In addition, several studies have reported that exposure to urban road dust has adverse health effects on the human respiratory system^14^. Our previous study also indicated a close relationship between cytotoxicity and several heavy metal compositions as well as organic compounds in road dust^15^. In this study, we further extended the analysis to include more sampling sites with greater variety covering a broader geographic area. We hypothesized that correlation between cytotoxicity and various chemical components as seen in the previous study^15^ could be observed similarly from other urban road dust which had been exposed to different chemical environments. Although many studies^16–18^ have revealed correlations between a single type of chemical component and health risks, few studies have provided collective impacts from various types of hazardous constituents in urban road dust. This study included various chemical components as a whole that could potentially affect cytotoxicity collectively and conducted an exploratory factor analysis to identify chemical constituents attributing to cytotoxicity.

The purpose of this study is to analyze the chemical composition of road dust and to investigate whether these properties might be related to the surrounding environment, including air quality and local pollution sources as well as to potential human health impact. The water-extractable components present in the re-suspended fine fractions of the road dust were compared among the 28 sampling sites in an effort to discover possible distinctions between their origins based on the fluorescent constituents. The cytotoxicity of urban road dust from 28 sites was analyzed, which is expected to provide a relationship between the chemical composition of urban road dust and human cells.

## Material and methods

### Sample preparation

#### Road dust sampling

The three largest emission sources each of CO, NH_3_, NO_x_, PM_2.5_, SO_x_, and volatile organic compounds (VOCs) were selected based on Korea air pollutant emission survey results in 2015, which included 13 different types of emission sources (Table S1) in total. Then, we selected the top three emission cities for each of 13 types of emission sources. Eleven overlapping cities were excluded from the 39 cities (top 3 cities * 13 emission sources), and the 28 different cities were selected for sampling sites. The geographic characteristics of the 28 sampling points are summarized in Table S2, and the corresponding locations are depicted in Figure S1. We followed the same procedures as those described by Koh and Kim^15^ for road dust sampling and pretreatment prior to chemical or cytotoxicological tests. The road dust sample for each site was composed of dust from 3 spots in the vicinity of vehicle roads or pedestrian roads collected by using a vacuum cleaner (GAS 14.4 V-Li, Bosch, Gerlingen, Germany) as described in the previous study^15^. The collected dust composite samples were placed in a polyethylene zipper bag^19,20^ for homogenization and then stored in an ice box where the temperature was kept at 0 ℃ during 3 days of field sampling. Within 24 h after field sampling, the composite dust samples from the 28 sites were sieved through a 100 μm screen and dried in a desiccator at room temperature for 24 h. The dried samples were placed in glass jars and stored at 2 °C before further treatment.

#### Size fractionation of the composite road dust

To a 100 mL Pyrex media bottle was added 0.5 g of the sieved composite road dust (< 100 μm), and the bottle was closed with a threaded cap with two Luer ports. The experimental setup used for dust particle size fractionation is depicted in Figure S2. The gas inlet was exposed to ambient air, and the inlet port airway was extended to the bottom of the media bottle using Norprene tubing to enable efficient resuspension of the dust. The gas outlet was connected to a portable pump (Leland Legacy pump, SKC Ltd., Dorset, UK), and ambient air was pulled into the media bottle to produce a turbulent dust plume. The re-suspended light particles were delivered to the gas outlet, and a Sioutas personal cascade impactor (SKC Ltd.) was used to fractionate the fine dust into four size compartments, RPM_2.5_, RPM_1_, RPM_0.5_, and RPM_0.25_, at a flow rate of 9 L min^−1^^21^.

### Characterization of the road dust solid samples

We also followed the same procedures as those described by Koh and Kim^15^ for C, H, N, and S elemental analysis. The sieved (< 100 μm) road dust samples from the 28 sampling sites were sent to the Center for Research Facilities of Chungnam National University, Republic of Korea, for C, H, N, and S elemental analysis using a Flash 2000 (Thermo Fisher Scientific, Inc., Waltham, MA, USA) organic elemental analyzer with a thermal conductivity detector. Three samples from each site were sent, and each sample was analyzed in triplicate, and the relative standard deviation of carbon concentrations for each triplicate measurement ranged between 1.4–16.7%. Because of the high relative standard deviations of H, N, and S concentrations, we did not use H, N, and S concentration for further exploratory analysis. The precision of this instrumental analysis was 0.1–0.3%, and the measuring range (100 ppm–100%) covered the carbon concentrations of all measured samples. For quality assurance, blank samples (empty tin foils) and standard samples were included at the beginning and the end of the sample analysis. The analysis results are summarized in Table S3.

The metal composition (wt%) analysis of the composite road dust samples was performed in triplicate using a ZSX Primus II (Rigaku, Japan) wavelength dispersive X-ray fluorescence (WD-XRF) analyzer. The ZSX Primus II was capable of analyze elements ^4^Be–^92^U, and it utilized a Rh anode X-ray tube (4 kW) and a LiF(220) crystal with scintillation counter to obtain K-line spectra. A semi-quantitative analysis was conducted by using SQX program with internal sensitivity library and subtracting background from each measurement. The full WD-XRF analysis results of the elemental compositions and the corresponding detection limits are summarized in Table S4.

The organic compounds in the solid road dust were qualitatively analyzed by pyrolysis–gas chromatography-mass spectrometry (py-GC–MS) using a PY-2020D pyrolyzer (Frontier Laboratories Ltd., Japan) connected to 5973 N mass selective detector (Agilent Technologies, USA). The samples (1–5 mg) were heated to 600 °C for 1 min, and the evolved gas was analyzed by GC–MS with the oven temperature increasing at a rate of 10 °C min^−1^ from 50 to 320ºC. The compounds corresponding to prominent peaks were selected from the NIST 17 mass spectral library according to the matches with the highest probability among candidate compounds with above 90% matching quality. The compounds were grouped into 4 groups—aliphatic hydrocarbons, aromatic hydrocarbons, phenol derivatives, and oxidized hydrocarbons. The sum of peak areas (a.u.) was divided by the sample mass (mg) for normalization. Although we made use of the numerical values for peak areas for further analysis, we did not intend to take these relative abundances as absolute amount. Because the detection sensitivity of each organic compound varies, the sum of peak areas could only be used for qualitative comparison between samples of similar matrices. The py-GC–MS spectra are shown in Figure S3, and the list of compounds corresponding to each group is summarized in Table S5.

### Characterization of the water-extractable composition of road dust solid samples

#### Total organic carbon (TOC) analysis

To 1 g of each sieved composite road dust sample (< 100 μm) was added 10 mL MilliQ water, and the slurry was sonicated at 25°C^22,23^ for one hour. A Bransonic CPX5800H-E ultrasonic bath (Branson Ultrasonics Corporation, Danbury, CT, USA) was used for sonication. The samples were prepared in triplicate. The extract was filtered through a 0.45 μm PTFE syringe filter to remove particulate matter. The filtered extract was stored at – 40 °C until further analysis. The samples were sent to the Center for Research Facilities at Chungnam National University, Republic of Korea, and were characterized using a Vario TOC Cube (Elementar, Langenselbold, Germany) based on a non-purgeable organic carbon (NPOC) analysis method. Each sample was measured three times, and the relative standard deviations of each triplicate ranged between 3–20%. The detection limit was 3 μg L^−1^, and the measuring range (0–25,000 mg L^−1^) covered the TOC concentrations of all measured samples. For quality assurance, standard samples were analyzed at the beginning of the sample analysis, and blank samples (deionized water) were included after every 9 to 18 measurements.

#### Fluorescence excitation-emission matrix (EEM) analysis

The water-extractable components present in the four sub-micrometer particle compartments (RPM_2.5_, RPM_1_, RPM_0.5_ and RPM_0.25_) were analyzed to obtain fluorescence EEMs. The size-fractionated fine dust samples collected on the filter papers were extracted using 5 mL MilliQ water by sonicating at 25°C^22,23^ for one hour. A Bransonic CPX5800H-E ultrasonic bath was used for sonication. The extract was filtered through a 0.45 μm PTFE syringe filter to remove particulate matter. The filtered extract was stored at − 40 °C until further analysis. A total of 112 samples from the re-suspended RPM_2.5_, RPM_1_, RPM_0.5_ and RPM_0.25_ fractions obtained from the 28 sampling sites were analyzed by a fluorescence EEM method.

The fluorescence EEMs were obtained using an RF6000 fluorescence spectrometer (Shimadzu, Japan) with a xenon lamp and photomultiplier tubes (PMT) voltage at 550 V. The filtered extract of the size-fractionated road dust was placed in a 1 cm quartz cuvette to obtain the emission spectra between 290 and 540 nm in 1 nm increments in response to excitation wavelengths between 250 and 400 nm at 5 nm increments. The precision of the excitation or emission wavelength was ± 1.0 nm, and the peak to peak signal to noise ratio (S/N) was greater than 350. The slit size was set to 5 nm for both the emission and excitation wavelengths, the response time was 0.5 s, and the scan speed was fixed at 2,400 nm min^−1^. The potential influence of metal ion complexation on the organic constituent EEM spectra was avoided by adjusting the sample pH to 1–2, which exchanged organic-bound metal ions with protons, prior to conducting the EEM measurements. The inner filter effect was eliminated from the fluorescence measurements by diluting all samples by one to sixfold using Milli-Q water (MilliporeSigma, Burlington, MA, USA) so that the UV absorbance at 254 nm was less than 0.05. The fluorescence data files (*.fd3) were converted to ASCII files using LabSolutions RF software. For quality assurance, blank samples (Milli-Q water) were measured once every 10 samples, and the EEM of a blank solution was subtracted from each sample EEM.

### Cytotoxicity analysis

#### Materials

Human skin fibroblast (BJ) and lung fibroblast (WI-38) cells were obtained from American Tissue Type Collection (ATCC, Manassas, VA, USA). Eagle’s Minimum Essential Medium (EMEM), Dulbecco’s phosphate buffer saline (DPBS), fetal bovine serum (FBS), penicillin–streptomycin and gentamicin were purchased from Thermo Fisher Scientific, Inc. (Waltham, MA, USA). Ninety-six-well flat-bottom tissue culture plates were purchased from Corning (Corning, NY, USA). Millex 0.22 μm syringe filters were obtained from MilliporeSigma. Cell Counting Kit-8 (CCK-8) was purchased from Dojindo (Kumamoto, Japan). Dried urban road dust samples were UV sterilized overnight and suspended in ultrapure water (0.22 μm sterile filtered) at a concentration of 10 mg mL^−1^. The sieved dust samples (< 100 μm) were briefly sonicated (5 min at 47 kHz, Branson 3,210) before application to cells.

#### Preparation of lung and skin fibroblasts

BJ and WI-38 fibroblasts were grown in EMEM supplemented with 10%/1% penicillin/streptomycin and 0.1% gentamicin reagent solution and cultured at 37 °C in 5% CO_2_. BJ and WI-38 with passage number less than 5 were used for consistency. Before seeding on to 96-well cell culture plate for cytotoxicity experiments, cells were washed with DPBS twice and trypsinzed with 0.05% trypsin–EDTA (Thermo Fisher Scientific, Inc.) for 3 min.

#### Cytotoxicity test of urban road dust

BJ and WI-38 cells were seeded in 96-well plates at a density of 1 × 10^4^ cells/well. After 12 h of incubation, dust suspensions in water of various concentrations (0.01, 0.05, 0.1, 0.25, 0.5, 1, 1.5, and 2 mg mL^−1^) were applied to BJ and WI-38. After 72 h of exposure to road dust, the cells were washed twice with DPBS and incubated with 10% CCK-8 in EMEM at 37 °C for 1 h. Absorbance at 450 nm was measured using a SpectraMax M5^e^ multimode microplate reader (Molecular Devices, San Jose, CA, USA). The cytotoxicity of the urban road dust from the 28 sites was estimated from the relative decrease in absorbance compared to that of the non-urban road dust treated control.

### Chemometric analysis

#### PARAFAC analysis of the water-extractable fluorescent components in the size-fractionated road dust

PARAFAC analysis methods have been widely applied to fluorescence EEMs to classify the major constituents and origins of various forms of natural organic^12,13,24^. The red emission spectrum (fluorescence emitted at longer wavelengths) is attributed to humic substances, aromatic compounds, or conjugated chemical bonds^25,26^. Conversely, the blue emission spectrum (fluorescence emitted at shorter wavelengths) is attributed to saturated carbon species, such as aliphatic compounds or amino acids.

PARAFAC analysis was conducted using MATLAB 2017b (Mathworks, Natick, MA, USA) with the open-source Fluor toolbox (https://www.models.life.ku.dk) following Bro’s methods^27–29^. The PARAFAC analysis method is briefly explained in the supplementary information.

The water extracts of the re-suspended particulate matter, namely, RPM_2.5_, RPM_1_, RPM_0.5_ and RPM_0.25_ triplicate samples collected from 28 sampling points, were analyzed. A total of 112 EEMs from the four particle size compartments obtained from the 28 sampling sites were imported into the N-way toolbox to extract the principal components of the water-extractable organic matter present in urban road dust. The number of principle components was determined based on the core consistency and residual sum of squares; the core consistency dropped significantly at 4 components, and the residual sum of squares reached a quasi-minimal level at 3 components. The component scores were used to estimate the relative concentrations of the fluorescence groups corresponding to the three PARAFAC factors.

#### Principal component analysis (PCA) for site classification

PCA was performed using the *psych* package in the open-source language R i386 version 3.3.1. The PCA input data were composed of water-extractable components (PARAFAC components %C1, %C2, and %C3), the metal compositions of urban road dust (traffic and industrial emissions), and organic compounds (phenolic compounds, aliphatic HCs, and aromatic HCs). The PCA was performed using the principal function to restructure the sample data with respect to the three largest eigenvectors. The varimax-rotated principal components were computed using the *GPArotation* package.

## Results and discussion

### Cytotoxicity in relation to the chemical profiles of urban road dust (< 100 μm)

A previous study^15^ reported chemical profiles of road dust with organic compounds and metal contents, and the results showed positive correlations between total organic carbon (C (wt%)) and the metal contents originating from industrial or traffic emissions. These two major groups of chemical components have also been revealed to have strong potential relationships with skin and lung cell viability. Here, we tested a larger set of road dust samples covering a greater geographic variance within South Korea to verify the previous study results.

**Figure 1:**
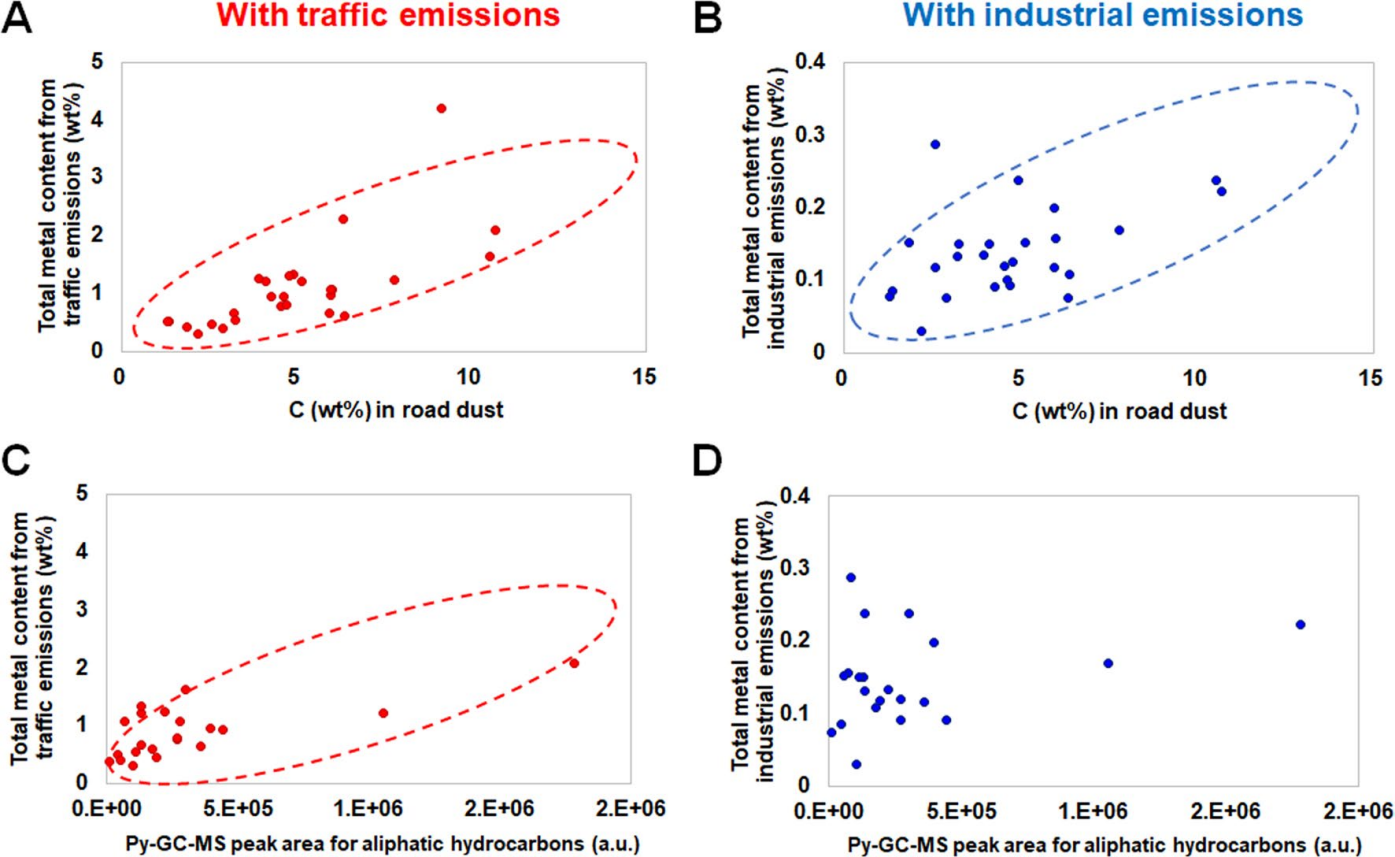
Correlations between total organic carbon and metal content originating from traffic emissions (**A**) or industrial emissions (**B**), and between py-GC–MS peak area for aliphatic hydrocarbons and metal content originating from traffic emissions (**C**) or industrial emissions (**D**) are depicted. The total carbon content (C (wt%)) and the sum of py-GC–MS peak areas corresponding to aliphatic hydrocarbons were used as proxies for the total organic carbon contained in the road dust samples.

Figure 1 confirms the positive correlation between the total organic carbon and metal content of road dust, and this study further specified a subset of organic compounds identified by a py-GC–MS method that may still exhibit a positive correlation with metal content. Notably, the total aliphatic hydrocarbon content identified by py-GC–MS analysis showed a positive correlation with the metal content originating from traffic emissions.

**Figure 2:**
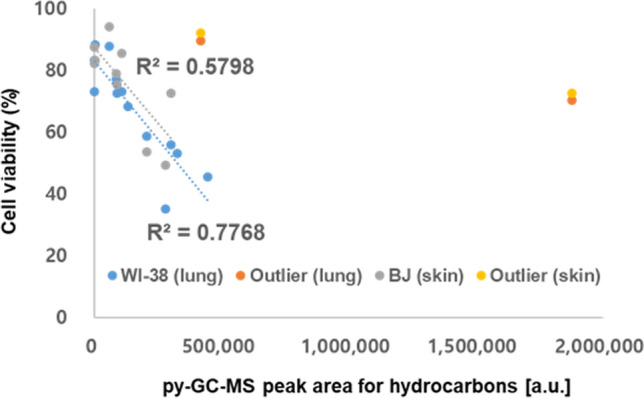
Correlations between cell viability (lung and skin) and abundance of total hydrocarbons in road dust. Two outliers each from the lung and skin cell viability results are depicted as separate groups against the rest of the data.

Koh and Kim^15^ employed a PCA method to test whether organic carbon contents (benzene derivatives and hydrocarbons) were associated with lung or skin cytotoxicity. We intended to investigate whether such correlations between organic carbon and cytotoxicity were maintained over various geographical sources. The dose–response relationship between urban road dust collected from the 28 different areas and WI-38 or BJ fibroblasts is depicted in Figure S4. Although the two fibroblasts originated from two distant organs (lung and skin), the cytotoxicity of urban road dust differed by less than 15% between fibroblast types at all concentrations of road dust particles we tested (Figure S4). Fifteen out of the 28 urban road dust samples induced less than 20% toxicity against the two cell lines at the highest concentration (2 mg/mL) we tested. Samples S2, S7 and S28 induced a > 50% decrease in BJ and WI-38 viability at 2 mg/mL. In addition, the micro-filtered portion of urban road dust did not induce > 10% toxicity in the cells we tested (Figure S5). The plot of the GC–MS peak area of hydrocarbons in urban road dust samples against cell viability showed that there is a correlation between the hydrocarbon content in urban road dust and BJ and WI-38 cytotoxicity (with R^2^ values of 0.5798 and 0.7768 for BJ and WI-38 cells, respectively, Fig. 2). These results, along with previous findings^15^, suggest that the content of hydrocarbons in urban road dust and metal contents are correlated with cytotoxicity in human skin and lung cells. To focus on more toxic and thus more problematic dust samples, we eliminated the cytotoxicity test results with greater than 90% lung cell viability. Two outliers with a much greater hydrocarbon abundance than cell viability were also removed; these outliers may have resulted from the inclusion of benign hydrocarbons in the corresponding samples. Because the carbon or hydrocarbon content was positively correlated with the total metal content from traffic or industrial emissions, as shown in Fig. 1, the negative correlation between cell viability and the relative abundance of hydrocarbons could partly originate from metals from traffic or industrial emissions that coexist with hydrocarbon compounds.

### Site classification based on the concentrations of organic compounds and the total metal content

Urban road dust contains various organic compounds originating from both anthropogenic and biogenic sources^30^. Organic compounds include PAHs, organic anions, and saturated hydrocarbons, such as n-alkanes, pristine, and phytane. PAHs originate from anthropogenic activities^31^, whereas organic anions and saturated hydrocarbons can be generated from either anthropogenic^32^ or biogenic sources^33^. There is no single analytical technique that can provide all speciation information for organic compounds, and several instrumental methods should be employed to obtain a comprehensive list of organic compounds. As a whole, this study analyzed a comprehensive set of organic compound groups, namely, total organic carbon, volatile or semi-volatile organic compounds, and water-extractable macromolecules, each of which may constitute an important dimension of chemical vector space. This chemical vector space could be combined with the metal content to yield extended chemical profiles that may vary significantly depending on different emission sources located near sampling sites.

Here, we further divided the organic carbon into four groups (aliphatic hydrocarbons, aromatic hydrocarbons, phenol derivatives, and oxidized hydrocarbons) using a py-GC–MS analysis method for the simplification of various chemical signatures. The compounds corresponding to these groups are listed in Table S5. Because the detection sensitivity of each organic compound varies, we did not translate the peak intensities (a.u.) as absolute amounts but as rough estimations for qualitative comparison.

**Figure 3:**
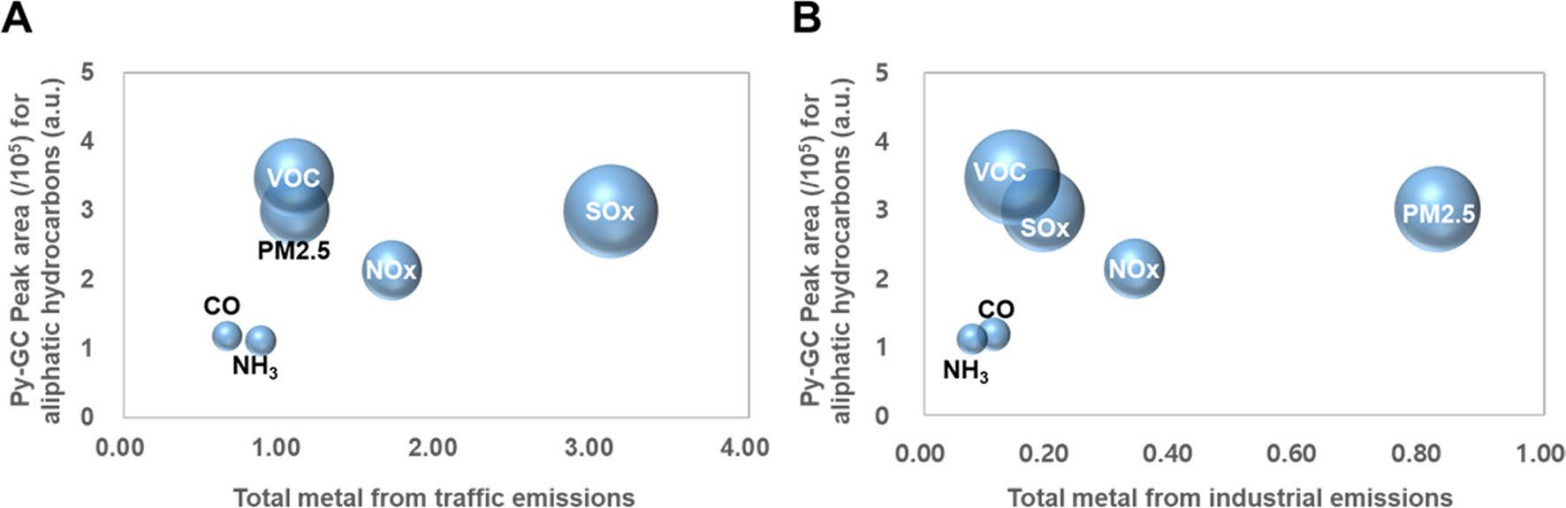
Correlation between the py-GC–MS peak area corresponding to aliphatic hydrocarbons and metal content originating from traffic (**A**) or industrial emissions (**B**). The radius of each data point corresponds to $\sqrt{{{\sigma }_{M}}^{2}+{{\sigma }_{HC}}^{2}}$, where ${\sigma }_{M}$ and ${\sigma }_{HC}$ represent the standard deviation of metal content and py-GC–MS peak area for aliphatic hydrocarbons, respectively.

Among the four groups of organic carbon classified in Table S5, the peak areas for aliphatic hydrocarbons had the greatest variance among the 28 sites. These wide ranges among the sampling sites may indicate various organic pollution sources influencing the local environment. Figure 3 exhibits chemical profiles based on the total metal content from traffic and industrial emissions and aliphatic hydrocarbons for samples from the 28 sampling sites; the profiles are classified into 6 distinctive groups depending on the key pollutants (CO, NH_3_, NO_x_, SO_x_, VOC, and PM_2.5_) from each site. The chemical profiles of the urban road dust consisted of the total py-GC–MS peak area and the metal content from traffic emissions (Fig. 3A) or industrial emissions (Fig. 3B). Both sets of groups in the chemical profiles distinguished the sampling sites with respect to several groups of key pollutants. This result may imply that the major air pollutants emitted in the vicinity could be used as proxies for various chemical environments surrounding fine road dust. However, the chemical profiles corresponding to (1) CO and NH_3_ groups or (2) SO_x_, VOC, and PM_2.5_ groups were relatively similar, thus providing a lower level of certainty in source identification. Therefore, a more extended chemical profile is required for more precise source identification.

### Site classification based on PARAFAC analysis of the water-extractable fluorescent components in size-fractionated road dust

Because of instrumental limitations, organic acids with high molecular weight could not be identified by py-GC–MS, and a suite of water-extractable macromolecules were analyzed by an excitation-emission matrix (EEM) method. Larger spectral areas of relatively high excitation–emission intensities in the EEMs indicated greater proportions of the corresponding fluorophores. Here, we extended the application of these methods to the classification of water-extractable organic matter present in urban road dust.

In this study, the PARAFAC analysis resulted in three PARAFAC components, C1, C2, and C3 that were major constituents of the water-extractable organic matter in urban road dust. The PARAFAC components (Figure S6) C1, C2 and C3 constituted 7–64%, 22–72%, and 12–47% of the fluorescence EEM spectra, respectively. The fluorescence EEMs for C1, C2, and C3 (Figure S6) revealed that the major components of the water-extractable organic matter in the urban road dust samples were saturated carbon species, such as aliphatic compounds or amino acids. Aryal et al*.*^34^ has reported that the major EEM components of water-extractable urban road dust were shown to be humic-like substances. Humic-like substances have distinct EEM spectra with fluorescence maxima around longer emission wavelengths compared with what we observed in this study. It was notable that the major fluorescent components extracted from the ultra-fine particulate matter we analyzed were close to low molecular weight organic matter originated from anthropogenic activity^34^.

**Figure 4:**
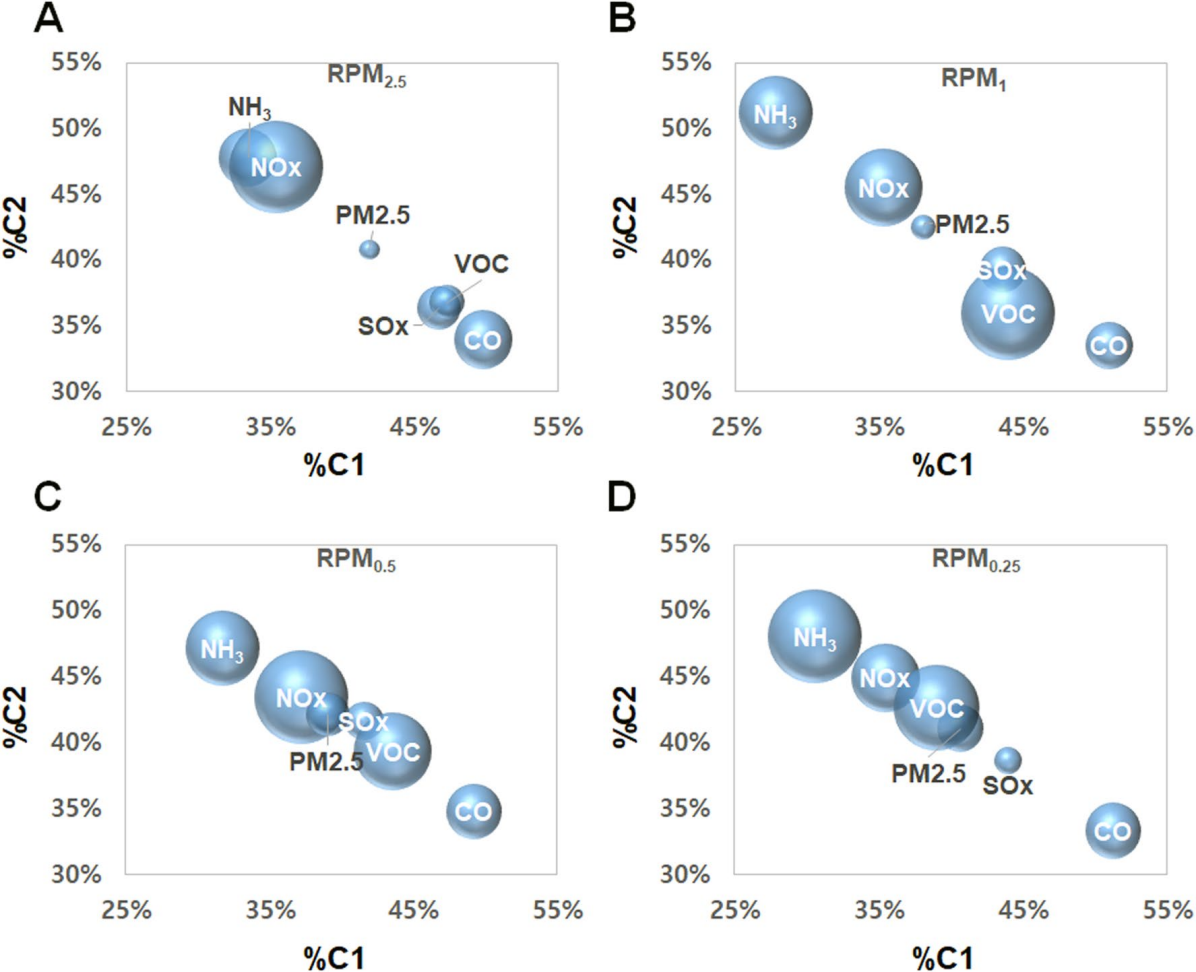
The 28 sampling sites grouped by the major pollutants (CO, NH_3_, NOx, PM_2.5_, SOx, and VOCs) were distinguishable by the relative compositions of the first two PARAFAC components (%C1 and %C2) contained in (**A**) RPM_2.5_, (**B**) RPM_1.0_, (**C**) RPM_0.5_, (**D**) RPM_0.25_. The radius of each data point corresponds to $\sqrt{{{\sigma }_{C1}}^{2}+{{\sigma }_{C2}}^{2}}$, where ${\sigma }_{C1}$ and ${\sigma }_{C2}$ represent the standard deviation of %C1 and %C2, respectively.

Figure 4 shows the distributions of the percent scores corresponding to the PARAFAC components C1 and C2 obtained from the RPM_2.5_ (50% cut-off point of 2.5 µm in terms of aerodynamic diameter), RPM_1_, RPM_0.5_ and RPM_0.25_ samples, and the pairs of %C1 and %C2 were clustered with respect to the 6 key air pollutants (CO, NH_3_, NO_x_, SO_x_, VOC, and PM_2.5_). Overall, the different key air pollutants resulted in unique chemical profiles composed of %C1 and %C2, and the relatively coarse fraction (RPM_2.5_) exhibited the least separation among the 6 designated groups of sites. As shown in Fig. 4A–D, the clusters associated with CO and NH_3_ were most widely separated among the 6 air pollutant clusters. In contrast, the chemical profiles of %C1 and %C2 provided the least distinction between PM_2.5_ and NO_x_ or SO_x_ (for RPM_1_ and RPM_0.5_) or between PM_2.5_ and VOC (for RPM_0.25_). These results may indicate that fine road dust (RPM_2.5_, RPM_1_, RPM_0.5_ and RPM_0.25_) contains rich information about the water-extractable fluorophores that vary from site to site. Therefore, the percent scores (%C1 and %C2) of fluorescent compounds in fine road dust could be utilized as indicators to distinguish the origins of road dust exposed to different air pollutants. Previously, chemical profiles composed of the total py-GC–MS area and metal content (Fig. 3) were not adequate to distinguish road dust samples originating from sites emitting CO or NH_3_ as key pollutants from those originating from other sites with other key pollutants. In contrast, the percent scores (%C1 and %C2) of fluorescent compounds distinguished the sites emitting CO or NH_3_ as key pollutants. As a result, the chemical information obtained from py-GC–MS, XRF analysis and EEMs complemented each other well for site identification of urban road dust.

### Principal component analysis

Principal component analysis (PCA) is one of the most widely used exploratory analysis methods to reduce the complexity of a given set of variables and to investigate the correlation between multiple variables that often include redundant pairs. Here, we utilized a PCA method to select a group of chemical components that could be utilized for source identification of urban road dust. The major principal components (PCs) obtained from the PCA results could be used as a basis to explain the chemical space of urban road dust in the form of linear combinations of PCs.

The PCA was conducted using the experimental or analytical results of water-extractable components extracted from fine fractions (RPM_0.25_ and RPM_0.5_), the metal compositions of urban road dust originating from traffic and industrial emissions and subsets of organic compounds (phenolic compounds, aliphatic hydrocarbons, and aromatic hydrocarbons) identified by a py-GC–MS method. The input variables were selected after preliminary exploratory analysis to extract moderately correlated parameters and to remove redundant variables from all elemental contents and chemical groups from py-GC–MS or EEM analysis. The PCA input data are summarized in the Supplementary Information (Table S6).

**Figure 5:**
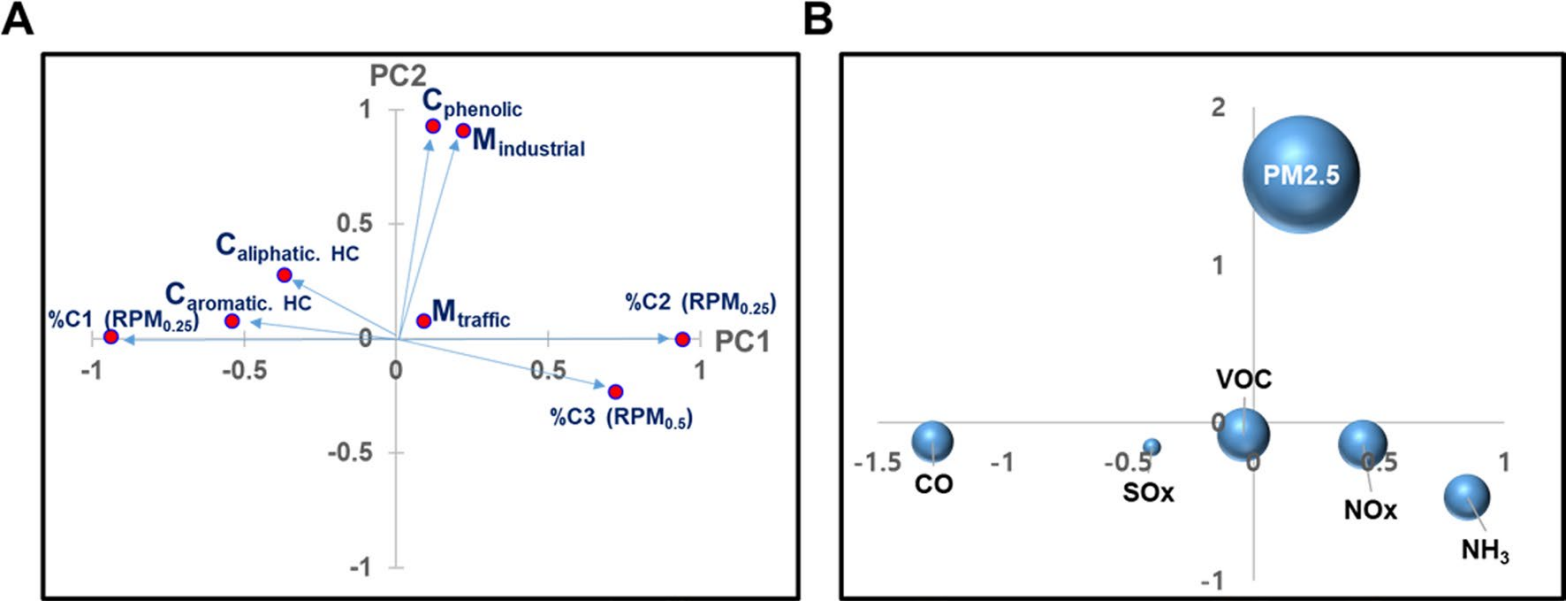
Principal component analysis results for source identification of road dust—(**A**): principal component (PC1 and PC2) score plots of major chemical constituents of fine road dust, (**B**) principal component (PC1 and PC2) score plots for chemical profiles of road dust originating from sites emitting CO, NH3, NOx, SOx, VOC, or PM2.5 as major pollutants. Each radius corresponds to $\sqrt{{{\sigma }_{PC1}}^{2}+{{\sigma }_{PC2}}^{2}}$, where ${\sigma }_{PC1}$ and ${\sigma }_{PC2}$ stand for the standard deviation of PC1 and PC2, respectively.

The PCA resulted in three principal components, PC1, PC2, and PC3, which explained approximately 49%, 33%, and 18% of the total variance, respectively. Therefore, linear combinations of PC1 and PC2 could describe 82% of the total variance of the input data. The loadings of PC1 and PC2 for each input parameter are depicted as vectors in Fig. 5A and show that the PARAFAC components of fluorescent organic matter (%C1 (RPM_0.25_), %C2 (RPM_0.25_), and %C3 (RPM_0.5_)) and hydrocarbon compounds (C_aliphatic. HC_ and C_aromatic. HC_) were most closely associated with each other. Similarly, the metal content originating from industrial emissions (M_industrial_) and phenolic compounds (C_phenolic_) showed a close correlation. Here, we used a set of PC1 and PC2 scores to simplify the complex chemical profiles of road dust into 2-dimensional space. The distribution of the scores (Fig. 5B) was clustered by the 6 key pollutants. Notably, the clusters corresponding to CO and NH_3_ were better separated than those classified by the abundance of aliphatic hydrocarbons and metal contents, as shown in Fig. 3. These results suggest that various chemical information about metal content, organic compounds, and fluorescent components provides a comprehensive basis for source identification of urban road dust. Further studies with more samples over longer periods of time are needed to verify whether these findings are generally applicable to wider geographical areas.

## Conclusions

This study analyzed chemical components contained in fine particles less than 100 μm or water extracts of fine re-suspended particles (RPM_2.5_, RPM_1_, RPM_0.5_ and RPM_0.25_) from fine urban road dust as potential indicators for potential human health impacts or source identification. This study was designed based on two assumptions: (1) the chemical composition of road dust affects human cell viability, and (2) major pollutants emitted from industrial facilities in the vicinity significantly contribute to the chemical profiles of fine urban dust. A total of 28 sites in the Korean Peninsula were chosen to include the top 3 emission spots for each of 13 types of emission sources contributing the most to air pollution by CO, NH_3_, NO_x_, PM_2.5_, SO_x_, and VOCs. We examined the chemical compositions of fine particles contained in road dust using various analytical methods, such as py-GC–MS, XRF and fluorescence EEM methods, and the array of analytical results was correlated with cytotoxicity or key pollutants from local sources.

We carried out dose–response tests to confirm positive correlations between total organic carbon (C (wt %)) and metal contents originating from industrial or traffic emissions. The road dust samples covered a greater geographic variance within South Korea than those used previously, enabling verification of previous study results. The negative correlation between cell viability and the relative abundance of hydrocarbons could partly originate from metals from traffic or industrial emissions that coexist with hydrocarbon compounds because the carbon or hydrocarbon content is positively correlated with the total metal content from traffic or industrial emissions. Therefore, the hydrocarbon contents and the amount of metals from traffic or industrial emissions could be used as proxies for potential human health impacts.

The elemental compositions, including the metal content, py-GC–MS peak areas of volatile or semi-volatile organic compounds, and water-extractable organic compounds and fluorescent species of size-fractionated re-suspended fine particulate matter, were also incorporated in the chemical profiles, which were tested as proxies for source identification of urban road dust. The metal content and aliphatic hydrocarbons provided relatively low levels of distinction between the CO and NH_3_ groups or the SO_x_, VOC, and PM_2.5_ groups. Clearer distinctions between sources were achieved when the chemical profile included analytical results from the fluorescence EEMs of water-extractable components. This result may imply that these analytical methods complemented each other well to compose chemical fingerprints for site identification of urban road dust.

A PCA method was employed to verify whether the chemical information obtained from py-GC–MS, XRF analysis and EEMs was sufficient for source identification. The PCA resulted in 3 principal components, and the two major PCs were used to further simplify the chemical information into a two-dimensional space. It was notable that the score distribution of PC1 and PC2 effectively distinguished the chemical profiles corresponding to different key pollutants. These results again suggest that various forms of chemical information about metal contents, organic compounds, and fluorescent components provide a comprehensive basis for source identification of urban road dust. However, further studies with more samples from wider geographical areas over longer periods of time are needed to generalize these findings.

## Supplementary information

Supplementary information.
