# Supplementary material for: Utilization of road dust chemical profiles for source identification and human health impact assessment
Source: Sci Rep. 2020 Aug 31;10:14259. doi: 10.1038/s41598-020-71180-x (PMC7459106; doi:10.1038/s41598-020-71180-x)
Supplement: Supplementary file 1 — Supplementary information. [file 41598_2020_71180_MOESM1_ESM.docx]

**Appendix A: Supplementary Information**

**Utilization of road dust chemical profiles for source identification and human health impact assessment**

Eun-Ah Kim^1,2*^, Byumseok Koh^3*^

^1^ Chemical Safety Research Center, Korea Research Institute of Chemical Technology

141 Gajeong-ro, Yuseong-gu, Daejeon 34114, Republic of Korea

^2^ National Assembly Futures Institute, Members Office Bldg, 1 Uisadang-daero, Yeongdeungpo-gu, Seoul 07233, Republic of Korea

^3^ Bio Platform Technology Research Center, Korea Research Institute of Chemical Technology

141 Gajeong-ro, Yuseong-gu, Daejeon 34114, Republic of Korea

* Corresponding authors

Eun-Ah Kim: Tel: +82-2-2224-9828; Fax: +82-2-786-3977; [eakim@nafi.re.kr](mailto:eakim@nafi.re.kr)

Byumseok Koh: Tel: +82-42-860-7465; Fax: +82-42-861-4246; bkoh@krict.re.kr

**Table of contents**

| **Tables and Figures** | |
| --- | --- |
| Table S1 | Top 3 emission sources CO, NH_3_, NO_x_, PM_2.5_, SO_x_, or volatile organic compounds (VOCs) |
| Table S2 | Geographic characteristics of the sampling sites. The average temperature and precipitation refer to the weekly averages up to the sampling date (S24 - S28: 31 August 2018, S1 - S7: 4 September 2018, S8 - S23: 5-7 September 2018). |
| Table S3 | Elemental (C, H, N, and S) compositions of the road dust samples sieved through a 100 µm screen. |
| Table S4 | Elemental compositions of the road dust obtained from the 7 sampling sites (ED-XRF analysis results) |
| Table S5 | The organic compounds identified by py-GC-MS and their corresponding groups |
| Table S6 | The input data for a PCA with water-extractable components (PARAFAC components %C1 (RPM_0.25_), %C2 (RPM_0.25_), %C3 (RPM_0.5_)), the metal compositions of urban road dust (traffic and industrial emission, M_traffic_ and M_industrial_, respectively), and organic compounds (phenolic compounds (C_phenolic_), aliphatic hydrocarbon (C_aliphatic,HC_), aromatic hydrocarbon (C_aromatic.HC_)) obtained from the 28 sampling sites |
| Figure S1 | Locations of the road dust sampling points (S1 –S28) corresponding to the GPS coordinates. |
| Figure S2 | Experimental setup used for dust particle size fractionation |
| Figure S3 | Py-GC-MS spectrum of each solid road dust sample from site S1-S28 |
| Figure S4 | Dose-response relationship between urban road dust samples collected from 28 different areas and lung (WI-38) and skin (BJ) fibroblasts viability. |
| Figure S5  Figure S6 | Dose-response relationship of water-extract part of urban road dusts collected from 28 different area and WI-38, BJ fibroblasts viability.  The three PARAFAC components that consist 112 EEMs obtained from water-extract of ultra-fine road dust |

**Tables and Figures**

**Table S1. Top 3 emission sources of CO, NH_3_, NO_x_, PM_2.5_, SO_x_, or volatile organic compounds (VOCs)**

| **Air pollutants** | **CO** | **NO_x_** | **SO_x_** | **PM_2.5_** | **VOC** | **NH_3_** |
| --- | --- | --- | --- | --- | --- | --- |
| **The greatest emission source** | Open burning agricultural debris | Truck | Public power plant | Open burning agricultural debris | Painting facility | Manure treatment |
| **The 2^nd^ greatest emission source** | Automobile | Vessel | Petroleum product industry | Re-suspended road dust | Miscellaneous use of organic solvent | Petroleum product industry |
| **The 3^rd^ greatest emission source** | Vessel | Construction equipment | Vessel | Vessel | Food and beverage processing | Fertilized farmland |

**Table S2. Geographic characteristics of the sampling sites. The average temperature and precipitation refer to the weekly averages up to the sampling date (S24 - S28: 31 August 2018, S1 - S7: 4 September 2018, S8 - S23: 5-7 September 2018).**

| Site index | GPS coordinate | Average temperature  (ºC) | Average precipitation  (mm) | Average PM_10_  (µg m^-3^) |
| --- | --- | --- | --- | --- |
| S1 | 37 º 33' 13" N  126 º 58' 10" E | 24.7 | 21.5 | 23.0 |
| S2 | 37 º 44' 47" N  127 º 2' 52" E | 24.1 | 7.4 | 26.1 |
| S3 | 37 º 45' 24" N  126ͦ º 46' 28" E | 24.1 | 7.4 | 26.1 |
| S4 | 37 º 41' 8" N  126 º 48' 51" E | 24.1 | 7.4 | 26.1 |
| S5 | 37 º 29' 43" N  126 º 29' 20" E | 24.8 | 14.7 | 26.9 |
| S6 | 37 º 27' 28" N  126 º 43' 40" E | 24.8 | 14.7 | 26.9 |
| S7 | 37 º 19' 21" N  126 º 49' 56" E | 24.1 | 7.4 | 26.1 |
| S8 | 35 º 58' 25" N  126 º 39' 14" E | 24.3 | 45.8 | 22.7 |
| S9 | 37 º 48' 18" N  126 º 53' 28" E | 24.3 | 45.8 | 22.7 |
| S10 | 35 º 34' 11" N  126 º 51' 22" E | 24.3 | 45.8 | 22.7 |
| S11 | 34 º 59' 26" N  126 º 28' 52" E | 24.6 | 41.4 | 17.0 |
| S12 | 34 º 34' 21" N  126 º 35' 56" E | 24.6 | 41.4 | 17.0 |
| S13 | 34 º 51' 11" N  127 º 43' 41" E | 24.6 | 41.4 | 17.0 |
| S14 | 34 º 56' 30" N  127 º 45' 29" E | 24.6 | 41.4 | 17.0 |
| S15 | 34 º 58' 23" N  128 º 19' 18" E | 24.7 | 11.1 | 32.6 |
| S16 | 37 º 51' 57" N  128 º 41' 30" E | 24.7 | 11.1 | 32.6 |
| S17 | 35 º 5' 37" N  128 º 51' 23" E | 24.0 | 30.2 | 21.4 |
| S18 | 35 º 26' 16" N  129 º 20' 15" E | 23.5 | 3.1 | 18.2 |
| S19 | 35 º 30' 11" N  129 º 25' 8" E | 23.5 | 3.1 | 18.2 |
| S20 | 35 º 30' 58" N  129 º 21' 32" E | 23.5 | 3.1 | 18.2 |
| S21 | 35 º 50' 17" N  128 º 29' 24" E | 23.4 | 10.3 | 24.2 |
| S22 | 36 º 6' 28" N  128 º 55' 9" E | 22.9 | 17.1 | 26.8 |
| S23 | 36 º 24' 37" N  128 º 9' 34" E | 22.9 | 17.1 | 26.8 |
| S24 | 37 º 11' 50" N  127 º 4' 20" E | 24.1 | 7.4 | 26.1 |
| S25 | 36 º 58' 27" N  126 º 50' 26" E | 24.1 | 7.4 | 26.1 |
| S26 | 36 º 43'57" N  126 º 8' 20" E | 24.4 | 24.1 | 14.2 |
| S27 | 36 º 36' 2" N  126 º 39' 39" E | 24.4 | 24.1 | 14.2 |
| S28 | 36 º 19' 59" N  126 º 36' 50" E | 24.4 | 24.1 | 14.2 |

Table S3. Elemental (C, H, N, and S) compositions of the road dust samples sieved through a 100 µm screen. The average concentrations of triplicate samples for each site are listed, and the relative standard deviation of carbon concentrations ranged between 1.4 – 16.7%. The relative standard deviations of H, N, and S concentrations were above 100% for some samples. Therefore, we only used C (wt%) for further exploratory analysis.

| Site ID | C (wt %) | H (wt %) | N (wt %) | S (wt %) |
| --- | --- | --- | --- | --- |
| S1 | 4.123 | 0.336 | 0.046 | 0.000 |
| S2 | 4.269 | 0.438 | 0.143 | 0.000 |
| S3 | 3.205 | 0.387 | 0.092 | 0.026 |
| S4 | 1.350 | 0.171 | 0.000 | 0.000 |
| S5 | 2.582 | 0.220 | 0.015 | 0.000 |
| S6 | 5.969 | 0.813 | 0.355 | 0.000 |
| S7 | 4.539 | 0.528 | 0.187 | 0.000 |
| S8 | 5.998 | 0.946 | 0.384 | 0.000 |
| S9 | 6.378 | 0.697 | 0.248 | 0.000 |
| S10 | 2.877 | 0.377 | 0.114 | 0.000 |
| S11 | 6.333 | 0.754 | 0.154 | 0.046 |
| S12 | 4.717 | 0.592 | 0.256 | 0.000 |
| S13 | 4.918 | 0.423 | 0.000 | 0.000 |
| S14 | 10.534 | 0.797 | 0.214 | 0.000 |
| S15 | 7.786 | 0.766 | 0.172 | 0.000 |
| S16 | 4.784 | 0.399 | 0.130 | 0.000 |
| S17 | 9.121 | 0.854 | 0.206 | 0.000 |
| S18 | 2.574 | 0.307 | 0.064 | 0.730 |
| S19 | 10.686 | 1.174 | 0.402 | 0.000 |
| S20 | 5.144 | 0.498 | 0.108 | 0.000 |
| S21 | 3.236 | 0.351 | 0.000 | 0.000 |
| S22 | 4.632 | 0.554 | 0.232 | 0.026 |
| S23 | 5.955 | 0.473 | 0.090 | 0.000 |
| S24 | 1.848 | 0.234 | 0.000 | 0.000 |
| S25 | 2.197 | 0.185 | 0.000 | 0.000 |
| S26 | 1.296 | 0.260 | 0.000 | 0.000 |
| S27 | 3.936 | 0.209 | 0.000 | 0.092 |
| S28 | 5.986 | 0.718 | 0.257 | 0.032 |

**Table S4. Elemental compositions of the road dust obtained from the 7 sampling sites (ED-XRF analysis results)**

| Site ID | Concentration / Detection limit (wt %) | | | | | | | | | | | | | | | | | | | | | |
| --- | --- | --- | --- | --- | --- | --- | --- | --- | --- | --- | --- | --- | --- | --- | --- | --- | --- | --- | --- | --- | --- | --- |
|  | Na | Mg | Al | Si | P | S | Cl | K | Ca | Ti | Cr | Mn | Fe | Ni | Cu | Zn | Rb | Sr | Zr | Ba | Pb |  |
| S1 | 1.01 / 0.08889 | 1.52 / 0.01494 | 10.5 / 0.01326 | 42.1 / 0.01827 | 0.253 / 0.0038 | 0.8 /  NA | 0.146 / 0.02186 | 7.07 / 0.01095 | 17.3 / 0.01603 | 1.52 / 0.04603 | 0.0921 / 0.02622 | 0.279 / 0.02263 | 15.2 / 0.12651 | 0.0581 / 0.01624 | 0.113 / 0.01515 | 0.819 / 0.01315 | 0.0941 / 0.00958 | 0.208 / 0.0093 | 0.296 / 0.053 | 0.445 / 0.13795 | 0.134 / 0.02829 |  |
| S2 | 0.746 / 0.09204 | 1.29 / 0.01725 | 11.9 / 0.01558 | 45.9 / 0.02082 | 0.341 / 0.00488 | 0.699 / 0.00844 | 0.145 / 0.03166 | 7.3 / 0.01789 | 12.6 / 0.01804 | 1.6 / 0.04235 | 0.0907 / 0.03477 | 0.253 / 0.03019 | 14.8 / 0.16421 | 0 /  NA | 0.0848 / 0.01903 | 0.606 / 0.01706 | 0.109 / 0.01256 | 0.198 / 0.01197 | 0.211 / 0.01296 | 0 /  NA | 0.135 / 0.03521 |  |
| S3 | 0.78 / 0.04948 | 1.34 / 0.01297 | 15.7 / 0.01347 | 45.2 / 0.01741 | 0.242 / 0.00341 | 0.571 / 0.00493 | 0.0761 / 0.01646 | 9.49 / 0.01062 | 7.97 / 0.01135 | 1.72 / 0.03065 | 0.0844 / 0.01917 | 0.206 / 0.01701 | 15.2 / 0.09306 | 0.048 / 0.01127 | 0.0533 / 0.01039 | 0.407 / 0.00909 | 0.0914 / 0.00664 | 0.115 / 0.00655 | 0.279 / 0.03775 | 0.37 / 0.08699 | 0.0516 / 0.0198 |  |
| S4 | 1.31 / 0.05289 | 1.63 / 0.01645 | 12 / 0.01498 | 44.1 / 0.02028 | 0.162 / 0.00492 | 0.4 / 0.009 | 0.0734 / 0.03025 | 6.68 / 0.01717 | 14.7 / 0.01834 | 1.03 / 0.05269 | 0.085 / 0.02771 | 0.294 / 0.02741 | 16.1 / 0.13536 | 0 /  NA | 0.0572 / 0.0186 | 0.156 / 0.01638 | 0.347 / 0.06755 | 0.243 / 0.01164 | 0.446 / 0.01361 | 0 /  NA | 0.0768 / 0.03418 |  |
| S5 | 1.53 / 0.04725 | 1.36 / 0.01332 | 10.8 / 0.01276 | 48.6 / 0.01924 | 0.166 / 0.00376 | 0.537 / 0.00649 | 0 /  NA | 8.71 / 0.01249 | 15.2 / 0.01555 | 1.16 / 0.02899 | 0.0761 / 0.02481 | 0.21 / 0.02131 | 11 / 0.10603 | 0.0419 / 0.01424 | 0.0392 / 0.01405 | 0.217 / 0.01249 | 0.0836 / 0.00909 | 0.163 / 0.00893 | 0.133 / 0.05131 | 0 /  NA | 0 /  NA |  |
| S6 | 0.787 / 0.05251 | 1.48 / 0.01333 | 14.6 / 0.01326 | 40.3 / 0.01592 | 0.615 / 0.00376 | 0.492 / 0.00466 | 0.139 / 0.01322 | 6.44 / 0.01179 | 14.4 / 0.01209 | 1.66 / 0.02448 | 0.146 / 0.0181 | 0.427 / 0.12643 | 17 / 0.01835 | 0.0531 / 0.0113 | 0.0631 / 0.01081 | 0.474 / 0.00944 | 0.0728 / 0.00726 | 0.119 / 0.00697 | 0.325 / 0.03999 | 0.258 / 0.09963 | 0.0788 / 0.01971 |  |
| S7 | 1.36 / 0.05988 | 1.6 / 0.01425 | 13.1 / 0.0142 | 43.8 / 0.01852 | 0.232 / 0.00359 | 0.443 / 0.00561 | 0.0521 / 0.0199 | 9.09 / 0.01197 | 14.8 / 0.01498 | 1.51 / 0.03884 | 0.0781 / 0.02448 | 0.279 / 0.02029 | 12.7 / 0.09935 | 0.0416 / 0.01443 | 0.0727 / 0.01325 | 0.419 / 0.01153 | 0.104 / 0.00922 | 0.172 / 0.00911 | 0.0817 / 0.00959 | 0 /  NA | 0.127 / 0.02643 |  |
| S8 | 0.562 / 0.056 | 3.47 / 0.01601 | 14.3 / 0.014 | 42.8 / 0.01791 | 0.372 / 0.00387 | 0.453 / 0.00519 | 0.032 / 0.01865 | 5.95 / 0.00971 | 4.74 / 0.00979 | 1.94 / 0.02833 | 0.104 / 0.01711 | 0.596 / 0.13978 | 23.2 / 0.02016 | 0.0531 / 0.01393 | 0.0442 / 0.0124 | 0.437 / 0.01028 | 0.0722 / 0.00808 | 0.0944 / 0.0077 | 0.119 / 0.044 | 0.442 / 0.11915 | 0.143 / 0.02291 |  |
| S9 | 0.832 / 0.0535 | 1.63 / 0.01431 | 14.7 / 0.01434 | 47.5 / 0.01881 | 0.341 / 0.0042 | 0.889 / 0.00649 | 0.081 / 0.02096 | 8.73 / 0.01196 | 8.99 / 0.01386 | 2.21 / 0.03734 | 0.0715 / 0.02134 | 0.229 / 0.02057 | 12.5 / 0.11335 | 0.0363 / 0.01409 | 0.0326 / 0.01295 | 0.345 / 0.0141 | 0.0882 / 0.00884 | 0.173 / 0.00868 | 0.189 / 0.04738 | 0.467 / 0.1289 | 0 /  NA |  |
| S10 | 1.44 / 0.03728 | 0.886 / 0.0134 | 16 / 0.01395 | 51.5 / 0.0194 | 0.306 / 0.00408 | 0.469 / 0.00625 | 0.0681 / 0.01874 | 9.96 / 0.01198 | 6.51 / 0.01247 | 1.55 / 0.0342 | 0.075 / 0.02035 | 0.192 / 0.01855 | 9.91 / 0.10209 | 0 /  NA | 0.0429 / 0.01127 | 0.16 / 0.00947 | 0.0918 / 0.007 | 0.194 / 0.0068 | 0.195 / 0.03795 | 0.409 / 0.1053 | 0 /  NA |  |
| S11 | 0 / 0.09339 | 1.42 / 0.01251 | 13.4 / 0.01199 | 46.2 / 0.01613 | 0.193 / 0.00323 | 1.05 / 0.00456 | 0.0714 / 0.01262 | 6.53 / 0.0086 | 14.6 / 0.01203 | 1.37 / 0.02514 | 0.0767 / 0.01708 | 0.43 / 0.11932 | 12.3 / 0.01615 | 0 /  NA | 0.055 / 0.00908 | 1.8 / 0.0084 | 0.0635 / 0.00656 | 0.112 / 0.00644 | 0.055 / 0.03705 | 0 /  NA | 0.148 / 0.01874 |  |
| S12 | 0.88 / 0.04345 | 1.12 / 0.01251 | 14.7 / 0.01335 | 47.4 / 0.01741 | 0.44 / 0.00385 | 0.578 / 0.00518 | 0.0997 / 0.01616 | 7.37 / 0.00996 | 12.4 / 0.01287 | 1.37 / 0.03679 | 0.0599 / 0.02104 | 0.426 / 0.11903 | 12 / 0.01507 | 0.0323 / 0.01166 | 0.075 / 0.01061 | 0.299 / 0.00914 | 0.0817 / 0.0072 | 0.145 / 0.00715 | 0.134 / 0.00764 | 0.328 / 0.11593 | 0.0639 / 0.02026 |  |
| S13 | 0.871 / 0.06161 | 1.1 / 0.01358 | 11.3 / 0.01221 | 42.1 / 0.01642 | 0.258 / 0.00324 | 0.963 / 0.00566 | 0.173 / 0.01673 | 5.3 / 0.00942 | 13.8 / 0.01213 | 1.28 / 0.02932 | 0.179 / 0.02061 | 0.637 / 0.13746 | 20.4 / 0.02001 | 0.0601 / 0.01382 | 0.153 / 0.01175 | 0.549 / 0.00992 | 0.0596 / 0.00749 | 0.145 / 0.00729 | 0.161 / 0.04014 | 0.302 / 0.09761 | 0.0755 / 0.02267 |  |
| S14 | 0.051 / 0.02636 | 1.83 / 0.01687 | 12.3 / 0.0149 | 34.7 / 0.01708 | 0.32 / 0.00345 | 1.12 / 0.00624 | 0.101 / 0.0207 | 4.83 / 0.00973 | 11.8 / 0.01252 | 1.12 / 0.04231 | 0.138 / 0.02043 | 0.573 / 0.18855 | 29 / 0.01785 | 0.101 / 0.01802 | 0.114 / 0.01624 | 0.951 / 0.01328 | 0.0587 / 0.01009 | 0.116 / 0.00958 | 0.15 / 0.0076 | 0 /  NA | 0.0955 / 0.04739 |  |
| S15 | 1.59 / 0.07418 | 1.78 / 0.01534 | 12.6 / 0.01466 | 46 / 0.01955 | 0.293 / 0.00432 | 1.27 / 0.00702 | 0.2 / 0.02177 | 5.87 / 0.01101 | 8.95 / 0.01328 | 2.33 / 0.03941 | 0.131 / 0.02255 | 0.535 / 0.15403 | 16.9 / 0.02159 | 0.0393 / 0.01595 | 0.0973 / 0.0146 | 0.6 / 0.0124 | 0.051 / 0.01002 | 0.18 / 0.00955 | 0.121 / 0.01011 | 0.379 / 0.14705 | 0.114 / 0.02794 |  |
| S16 | 0.538 / 0.06593 | 1.76 / 0.01423 | 10.3 / 0.01198 | 33.4 / 0.0148 | 0.243 / 0.00306 | 0.454 / 0.00536 | 0.0583 / 0.01691 | 4.27 / 0.00832 | 31.8 / 0.01633 | 1.34 / 0.03362 | 0.0691 / 0.02522 | 0.607 / 0.1586 | 13.6 / 0.02136 | 0.0563 / 0.01532 | 0.147 / 0.01312 | 0.553 / 0.01133 | 0.0486 / 0.00901 | 0.187 / 0.00873 | 0.132 / 0.00943 | 0.326 / 0.13331 | 0.158 / 0.02595 |  |
| S17 | 0 / 0.15482 | 2 / 0.01536 | 11 / 0.01302 | 35.5 / 0.01698 | 0.259 / 0.00359 | 1.23 / 0.00663 | 0.0852 / 0.01962 | 2.99 / 0.00901 | 9.22 / 0.01139 | 1.75 / 0.02776 | 0.712 / 0.01941 | 1.13 / 0.01912 | 29.5 / 0.0163 | 0.294 / 0.01825 | 0.26 / 0.01603 | 2.8 / 0.01407 | 0.0427 / 0.0103 | 0.171 / 0.00979 | 0.395 / 0.05042 | 0 /  NA | 0.339 / 0.03045 |  |
| S18 | 0 / 0.15859 | 1.36 / 0.0202 | 11.9 / 0.01409 | 38 / 0.01737 | 0.171 / 0.00349 | 1.61 / 0.00849 | 0.00675 / 0.02012 | 4.08 / 0.01009 | 8.46 / 0.01138 | 1.04 / 0.03292 | 0.198 / 0.01861 | 0.904 / 0.11656 | 20.1 / 0.0152 | 0.0897 / 0.01474 | 1.69 / 0.01455 | 7.03 / 0.01404 | 0.0694 / 0.01018 | 0.214 / 0.0096 | 0.155 / 0.04934 | 1.37 / NA | 0.0867 / 0.09972 |  |
| S19 | 0.22 / 0.07416 | 2.06 / 0.01705 | 13.8 / 0.01638 | 45.5 / 0.02095 | 0.477 / 0.00489 | 1.73 / 0.00919 | 0.162 / 0.02694 | 5.08 / 0.01286 | 7.63 / 0.01419 | 2 / 0.04183 | 0.122 / 0.02933 | 0.49 / 0.0243 | 18.4 / 0.13994 | 0.101 / 0.01959 | 0.331 / 0.01879 | 1.27 / 0.01561 | 0.0627 / 0.01261 | 0.151 / 0.01261 | 0.165 / 0.01273 | 0 /  NA | 0.257 / 0.03576 |  |
| S20 | 1.5 / 0.06556 | 1.38 / 0.01372 | 11 / 0.01253 | 45.2 / 0.01743 | 0.267 / 0.00355 | 1.23 / 0.00588 | 0.108 / 0.01817 | 4.96 / 0.01203 | 15.1 / 0.01298 | 1.42 / 0.03171 | 0.107 / 0.0226 | 0.477 / 0.14394 | 14.8 / 0.01959 | 0.0448 / 0.0128 | 0.0909 / 0.01205 | 0.633 / 0.01005 | 0.0588 / 0.00801 | 0.303 / 0.00785 | 0.0712 / 0.00896 | 0.397 / 0.10032 | 0.153 / 0.02262 |  |
| S21 | 1.75 / 0.05079 | 2.5 / 0.01522 | 13.1 / 0.01443 | 46.8 / 0.0196 | 0.221 / 0.00393 | 0.561 / 0.00658 | 0 /  NA | 7.29 / 0.01211 | 10 / 0.01361 | 1.78 / 0.03295 | 0.0976 / 0.02452 | 0.221 / 0.02145 | 14.4 / 0.11949 | 0.053 / 0.01539 | 0.0526 / 0.01389 | 0.266 / 0.01124 | 0.0699 / 0.00863 | 0.277 / 0.0084 | 0.077 / 0.04796 | 0.411 / 0.12371 | 0.0603 / 0.02621 |  |
| S22 | 1.59 / 0.0584 | 1.54 / 0.01387 | 13.6 / 0.01367 | 48.4 / 0.01767 | 0.414 / 0.00383 | 0.775 / 0.00594 | 0 /  NA | 6.4 / 0.01035 | 11 / 0.01274 | 1.42 / 0.03397 | 0.066 / 0.02018 | 0.433 / 0.13524 | 13.2 / 0.01857 | 0.0338 / 0.0124 | 0.0445 / 0.01169 | 0.48 / 0.01011 | 0.116 / 0.0446 | 0.149 / 0.00769 | 0.109 / 0.00821 | 0 /  NA | 0 /  NA |  |
| S23 | 1.51 / 0.04878 | 2.12 / 0.01481 | 13.2 / 0.01389 | 44.2 / 0.01853 | 0.19 / 0.00348 | 0.807 / 0.00614 | 0.0507 / 0.02037 | 8.24 / 0.01141 | 16.7 / 0.01561 | 2.54 / 0.03735 | 0.0747 / 0.02544 | 0.339 / 0.14659 | 9.29 / 0.02008 | 0.0424 / 0.01387 | 0.0426 / 0.01271 | 0.267 / 0.01063 | 0.0777 / 0.00863 | 0.181 / 0.00851 | 0.122 / 0.00925 | 0 /  NA | 0 /  NA |  |
| S24 | 0.48 / 0.04952 | 0.748 / 0.01417 | 10.8 / 0.01476 | 59.8 / 0.02278 | 0.178 / 0.00478 | 0.551 / 0.00923 | 0 /  NA | 8.96 / 0.01537 | 2.86 / 0.01462 | 1.57 / 0.041 | 0.0963 / 0.02618 | 0.23 / 0.02367 | 12.6 / 0.13596 | 0.0564 / 0.01762 | 0 /  NA | 0.186 / 0.01308 | 0.0997 / 0.00996 | 0.109 / 0.00977 | 0.264 / 0.01015 | 0.389 / 0.14694 | 0 /  NA |  |
| S25 | 1.84 / 0.03309 | 0.953 / 0.01375 | 10.7 / 0.01262 | 49 / 0.01843 | 0.155 / 0.00413 | 0.478 / 0.00598 | 0.0461 / 0.0213 | 9.52 / 0.01204 | 14.9 / 0.01533 | 0.948 / 0.03802 | 0 /  NA | 0.227 / 0.0225 | 9.61 / 0.11532 | 0.0295 / 0.01413 | 0 /  NA | 0.0821 / 0.01122 | 0.118 / 0.00837 | 0.162 / 0.00822 | 0.105 / 0.00882 | 0.891 / 0.15121 | 0 /  NA |  |
| S26 | 0.828 / 0.02942 | 0.77 / 0.01285 | 11.1 / 0.01292 | 62.4 / 0.02076 | 0.339 / 0.00478 | 0.259 / 0.00666 | 0 /  NA | 8.75 / 0.01284 | 4.47 / 0.01327 | 1.36 / 0.03918 | 0.0775 / 0.02559 | 0.162 / 0.0196 | 8.98 / 0.11078 | 0 /  NA | 0.274 / 0.01243 | 0.0696 / 0.0104 | 0.0831 / 0.00767 | 0.0988 / 0.00753 | 0.133 / 0.04164 | 0 /  NA | 0 /  NA |  |
| S27 | 1.08 / 0.09778 | 1.5 / 0.01644 | 12.3 / 0.01517 | 46.7 / 0.02165 | 0.218 / 0.00402 | 0.844 / 0.00849 | 0.0401 / 0.02695 | 7.58 / 0.01401 | 8.81 / 0.01509 | 1.16 / 0.03603 | 0.0884 / 0.03018 | 0.279 / 0.02573 | 16.7 / 0.14187 | 0.046 / 0.01986 | 0.22 / 0.01757 | 0.756 / 0.01445 | 0.103 / 0.01116 | 0.239 / 0.01085 | 0.25 / 0.01227 | 0.483 / 0.16548 | 0.092 / 0.03356 |  |
| S28 | 0.831 / 0.05408 | 1.82 / 0.01667 | 15 / 0.01578 | 38.4 / 0.01907 | 0.59 / 0.00424 | 0.844 / 0.00709 | 0.894 / 0.02609 | 6.66 / 0.01194 | 6.24 / 0.01255 | 1.52 / 0.03853 | 1.7 / 0.02334 | 0.352 / 0.02289 | 23.5 / 0.02457 | 0.451 / 0.01692 | 0.36 / 0.01684 | 0.356 / 0.01365 | 0.106 / 0.01015 | 0.156 / 0.00997 | 0.183 / 0.05556 | 0 /  NA | 0 /  NA |  |

**Table S5. The organic compounds identified by py-GC-MS and their corresponding groups**

| Aliphatic hydrocarbon | Aromatic hydrocarbon | Phenol-derivative | Oxidized hydrocarbon |
| --- | --- | --- | --- |
| 1,2-Dimethylcyclopropene  1,3,5-Cycloheptatriene  1,3-Cyclohexadiene  1-Dodecene  1-Methyl-1H-indene  3-Methyl-1H-indene  1-UNDECENE  2-Methyl-Z-4-tetradecene  2-Methyl-2-penten-4-yne  3-Penten-1-yne  5-Methyl-5-vinyl-1,3-cyclopentadien  Bicyclo[4.2.0]octa-1,3,5-triene  4-Ethenyl-cyclohexene  1-Methyl-2-pentyl cyclopropane  Decane  Dicyclopropylacetylene  dl-Limonene  Ethynylcyclopentene  Hepta-1,3-diyne  Propane  Tricyclo[5.1.0.0(2,8)]oct-4-en  Z-.beta.-ocimene | 4-Phenyl-1-buten-3-yne  Benzene  (1-methylethenyl)-benzene  1,2-propadienyl-benzene  1,3-dimethyl-benzene,  1,4-dimethyl-benzene,  Propyl-benzene,  Ethylbenzene  Methylstyrene  Styrene  Toluene  unidentifiedC2-benzene  unidentifiedC3-benzene | Phenol  3,5-dimethyl-phenol,  3-methyl-phenol,  4,4'-(1-methylethylidene)b2phenol,  4-methyl-phenol, | (E)-Hex-2-en-4ynal  1,3-Isobenzofurandione  1-Methoxy-1-buten-3-yne  2-Furancarboxaldehyde  9,10-Dihydrofulvalene  Benzenemethanol  Cyclopropanecarboxamide  Hexadecanoicacid  1,2-dihydro-naphthalene,  Octadecanoicacid |

**Table S6. The input data for a PCA with water-extractable components (PARAFAC components %C1(RPM_0.25_), %C2(RPM_0.25_), %C3(RPM_0.5_)), the metal compositions of urban road dust (traffic and industrial emission, M_traffic_ and M_industrial_, respectively), and organic compounds (phenolic compounds (C_phenolic_), aliphatic hydrocarbon (C_aliphatic,HC_), aromatic hydrocarbon (C_aromatic.HC_)) obtained from the 28 sampling sites**

| Site index | M_traffic_ | M_industrial_ | C_aliphatic,HC_ | C_aromatic.HC_ | C_phenolic_ | %C1  (RPM_0.25_) | %C2  (RPM_0.25_) | %C3  (RPM_0.5_) |
| --- | --- | --- | --- | --- | --- | --- | --- | --- |
| S1 | 0.54 | 0.15 | 111,692 | 200,094 | 20,537 | 44.0% | 38.0% | 17.7% |
| S2 | 0.96 | 0.10 | 0 | 398,245 | 46,742 | 54.7% | 31.7% | 12.6% |
| S3 | 0.65 | 0.12 | 361,524 | 254,881 | 0 | 58.7% | 25.9% | 16.9% |
| S4 | 0.51 | 0.08 | 0 | 0 | 13,374 | 47.7% | 37.7% | 16.8% |
| S5 | 0.61 | 0.11 | 174,965 | 0 | 33,955 | 31.2% | 46.4% | 19.7% |
| S6 | 0.39 | 0.08 | 9,907 | 75,144 | 0 | 32.6% | 46.8% | 21.8% |
| S7 | 2.29 | 0.08 | 0 | 186,143 | 0 | 22.8% | 54.5% | 23.7% |
| S8 | 0.80 | 0.09 | 268,937 | 0 | 64,633 | 18.2% | 57.7% | 23.1% |
| S9 | 0.31 | 0.03 | 103,221 | 0 | 0 | 48.0% | 35.0% | 17.4% |
| S10 | 0.94 | 0.09 | 443,926 | 0 | 0 | 41.2% | 40.8% | 18.8% |
| S11 | 0.47 | 0.12 | 191,573 | 32,362 | 0 | 44.0% | 39.9% | 20.3% |
| S12 | 1.34 | 0.24 | 133,245 | 0 | 0 | 22.8% | 54.1% | 23.9% |
| S13 | 1.64 | 0.24 | 301,221 | 155,489 | 25,856 | 38.6% | 41.2% | 15.5% |
| S14 | 4.19 | 1.01 | 0 | 0 | 0 | 30.8% | 48.5% | 18.2% |
| S15 | 0.96 | 0.20 | 395,509 | 31,304 | 101,922 | 40.3% | 40.5% | 18.7% |
| S16 | 1.26 | 0.13 | 225,569 | 30,142 | 0 | 47.1% | 36.7% | 20.1% |
| S17 | 1.07 | 2.15 | 280,346 | 54,594 | 312,699 | 34.6% | 46.0% | 17.7% |
| S18 | 1.23 | 0.17 | 1,055,059 | 92,636 | 0 | 39.5% | 41.8% | 14.9% |
| S19 | 9.62 | 0.29 | 84,335 | 63,721 | 0 | 42.6% | 38.9% | 18.5% |
| S20 | 1.20 | 0.15 | 0 | 14,864 | 0 | 46.6% | 36.2% | 16.7% |
| S21 | 0.42 | 0.15 | 56,912 | 6,994 | 4,509 | 47.1% | 37.7% | 17.0% |
| S22 | 1.21 | 0.15 | 130,764 | 25,928 | 13,802 | 22.8% | 54.5% | 13.2% |
| S23 | 0.67 | 0.13 | 134,129 | 14,276 | 17,994 | 45.6% | 37.7% | 14.4% |
| S24 | 0.51 | 0.09 | 43,876 | 6,158 | 0 | 44.9% | 37.8% | 18.5% |
| S25 | 0.77 | 0.12 | 271,481 | 26,722 | 0 | 35.3% | 46.7% | 19.3% |
| S26 | 1.08 | 0.16 | 70,779 | 0 | 73,711 | 37.0% | 44.6% | 20.5% |
| S27 | 1.31 | 0.13 | 0 | 0 | 0 | 33.3% | 45.8% | 18.4% |
| S28 | 2.09 | 0.22 | 1,781,754 | 98,914 | 69,198 | 54.5% | 31.4% | 15.5% |


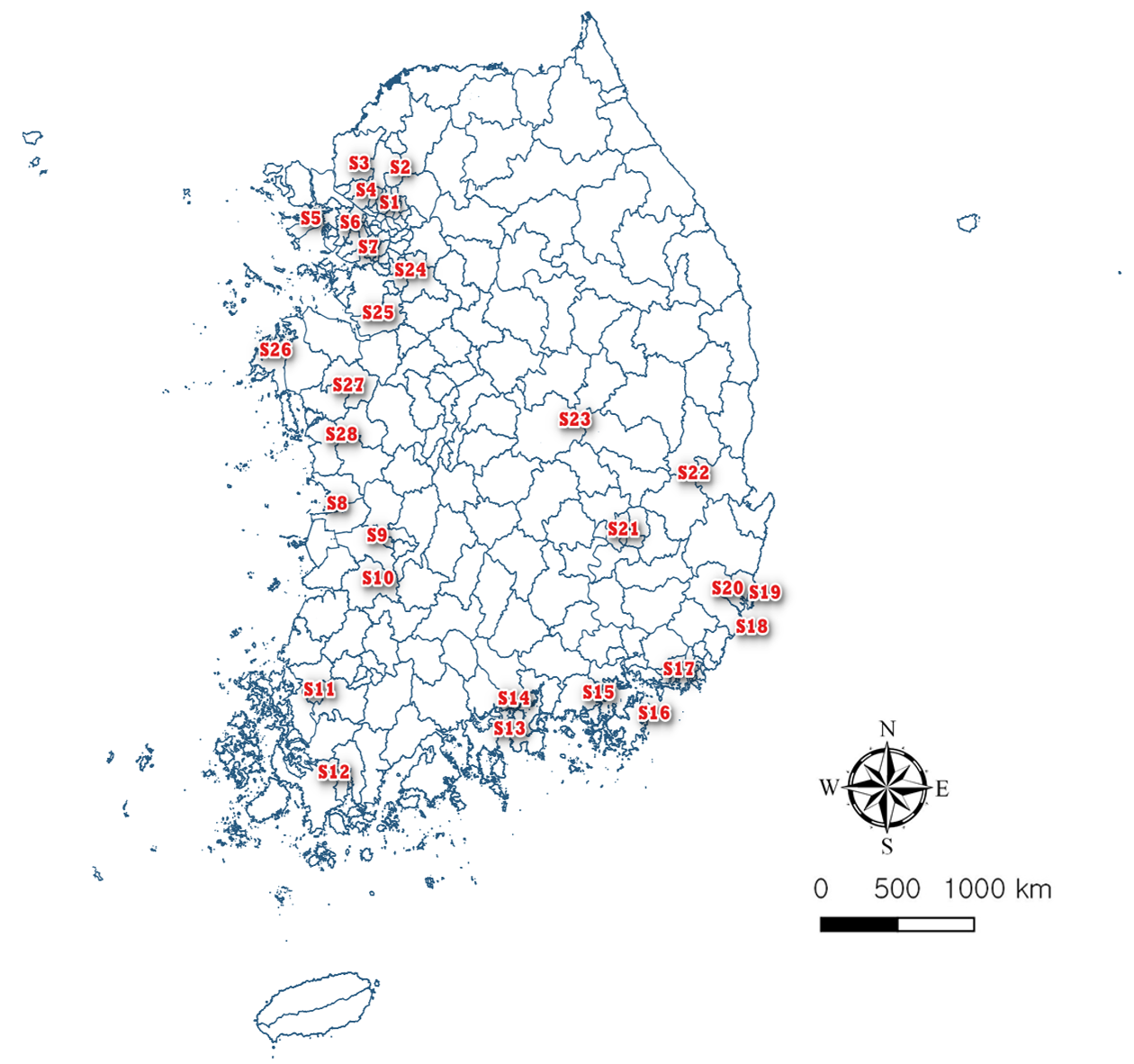


**Figure S1. Locations of the 28 road dust sampling points (S1 – S28) corresponding to the GPS coordinates.**


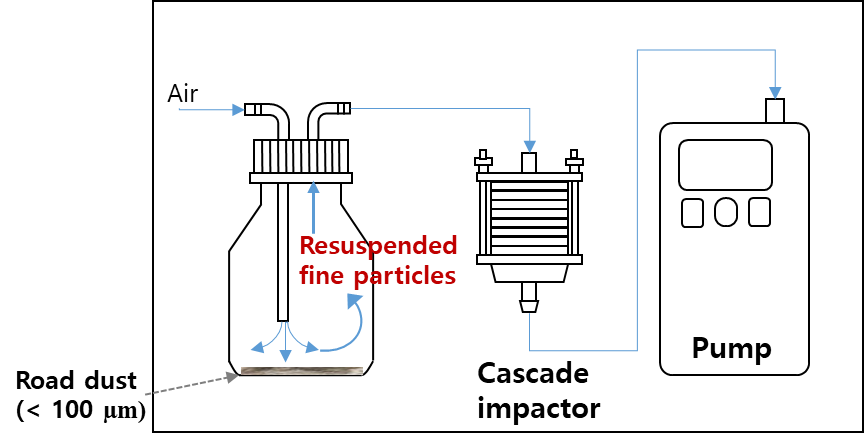


**Figure S2. Experimental setup used for dust particle size fractionation**

| S1  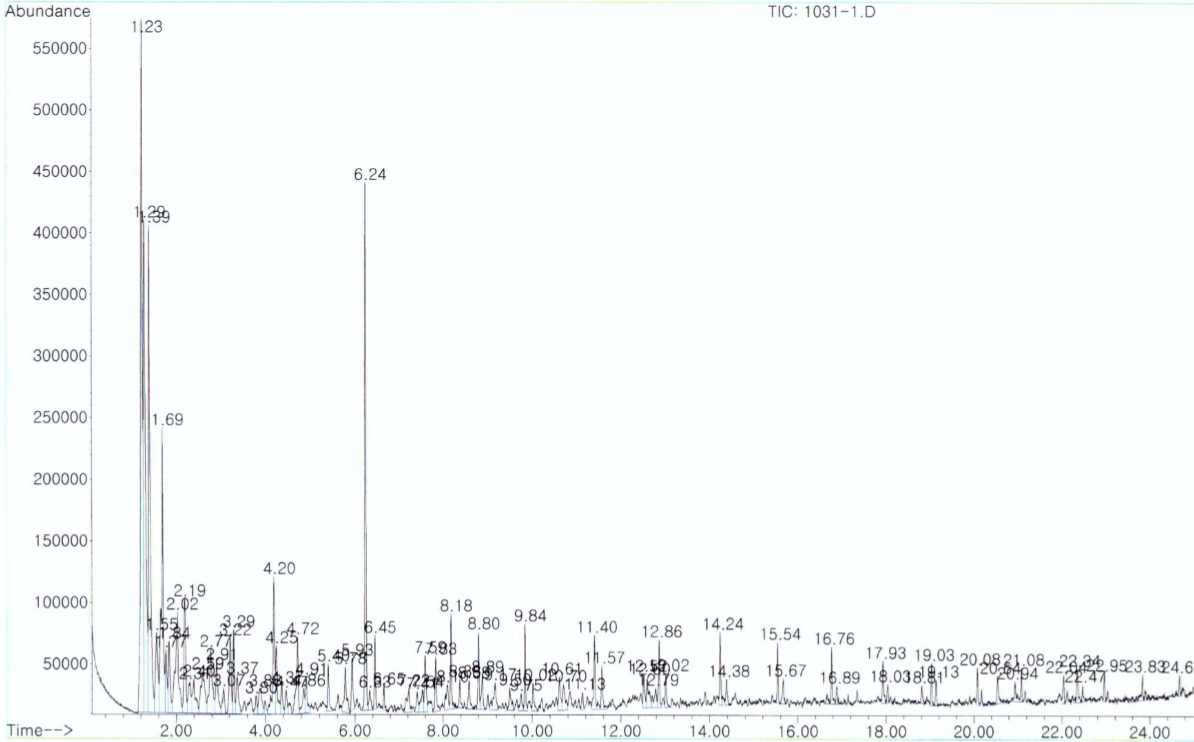 | S2  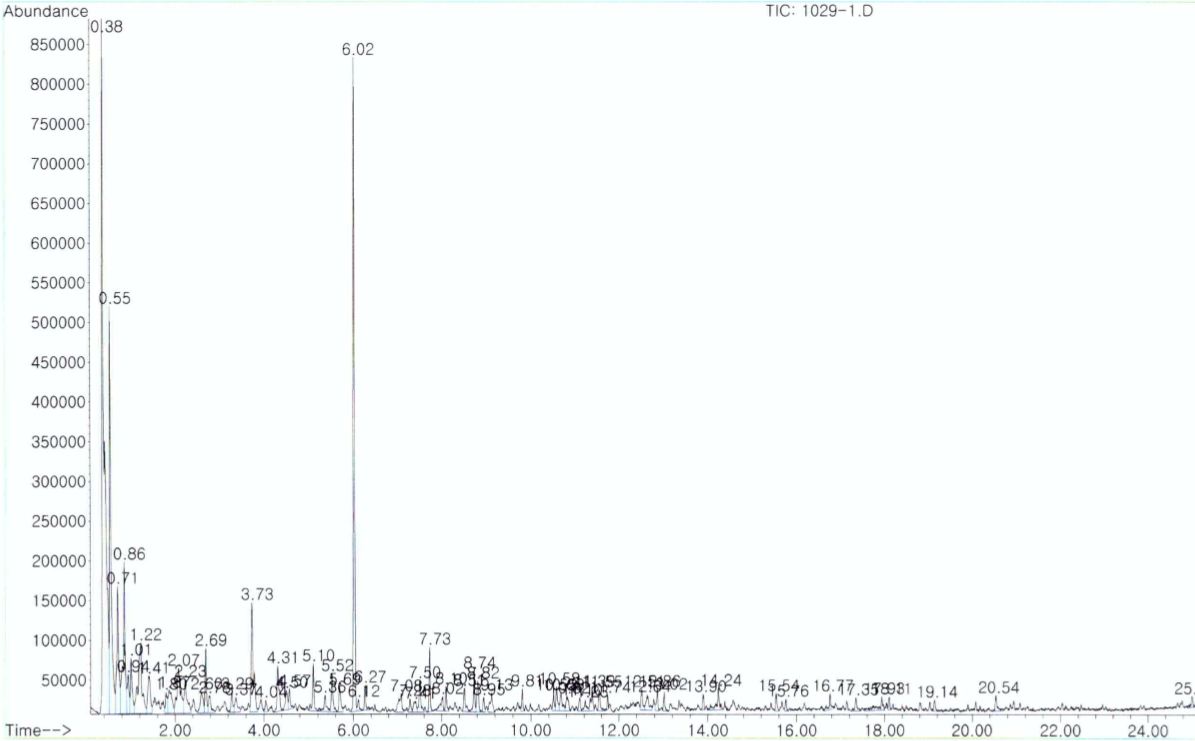 |
| --- | --- |
| S3  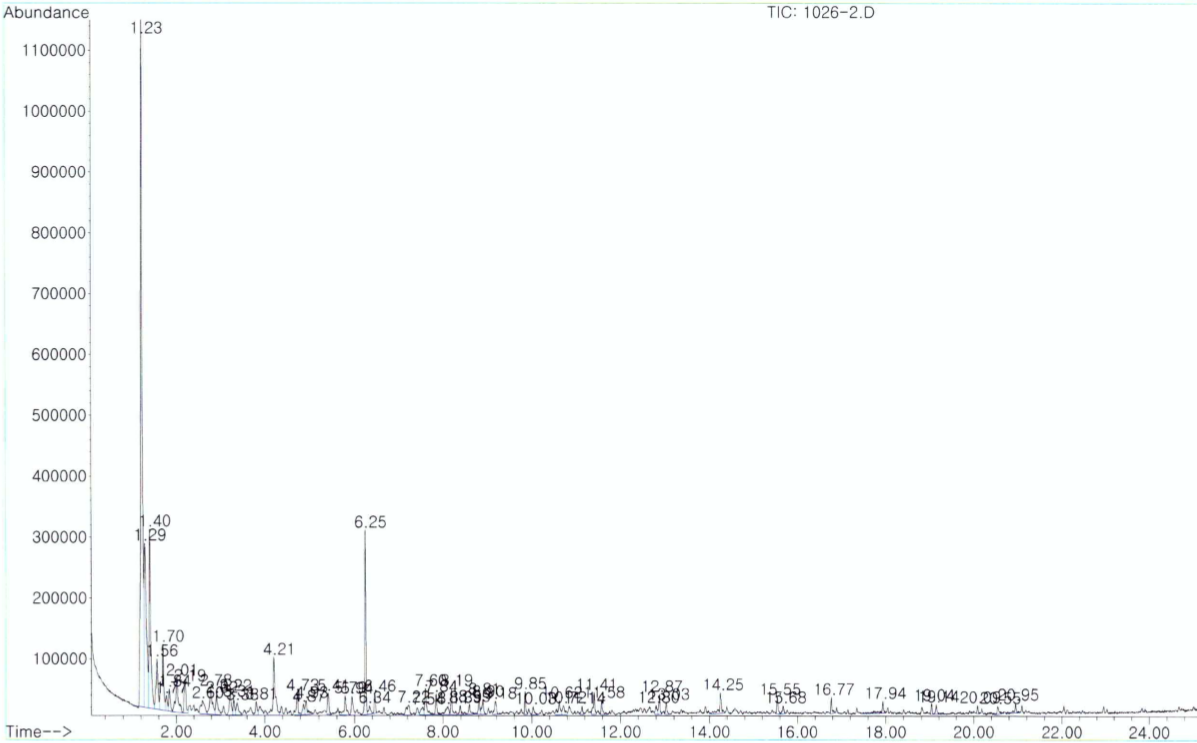 | S4  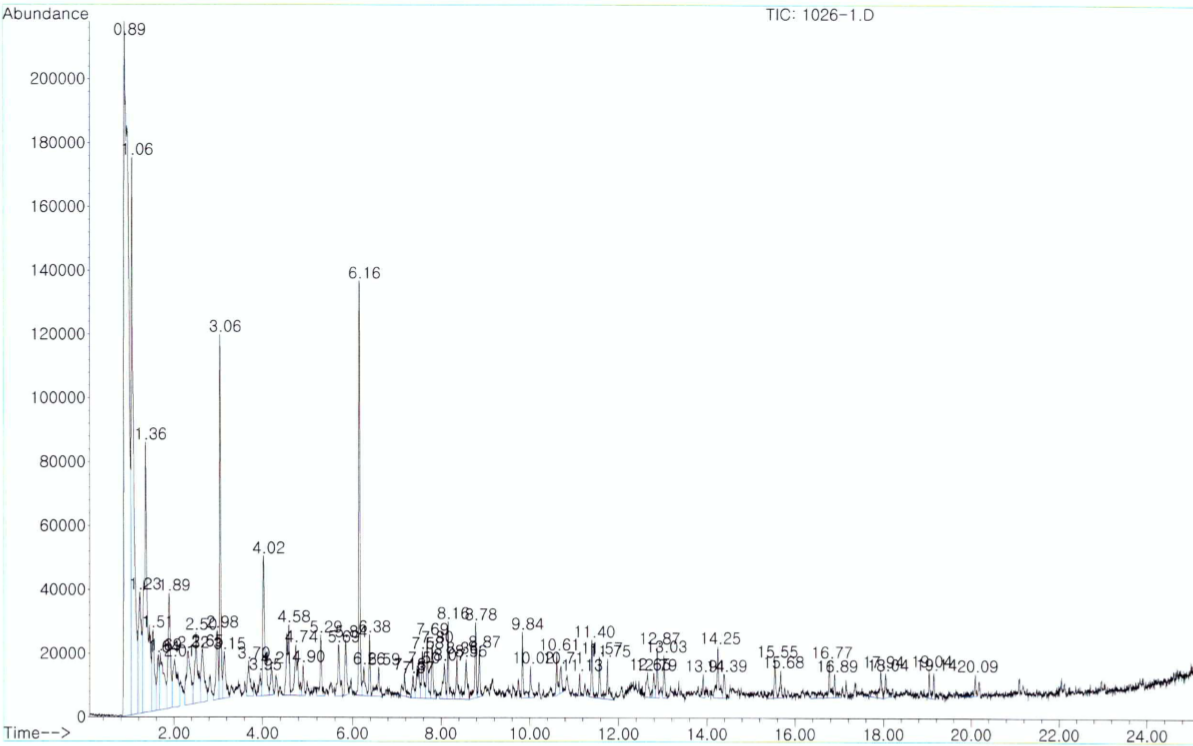 |
| S5  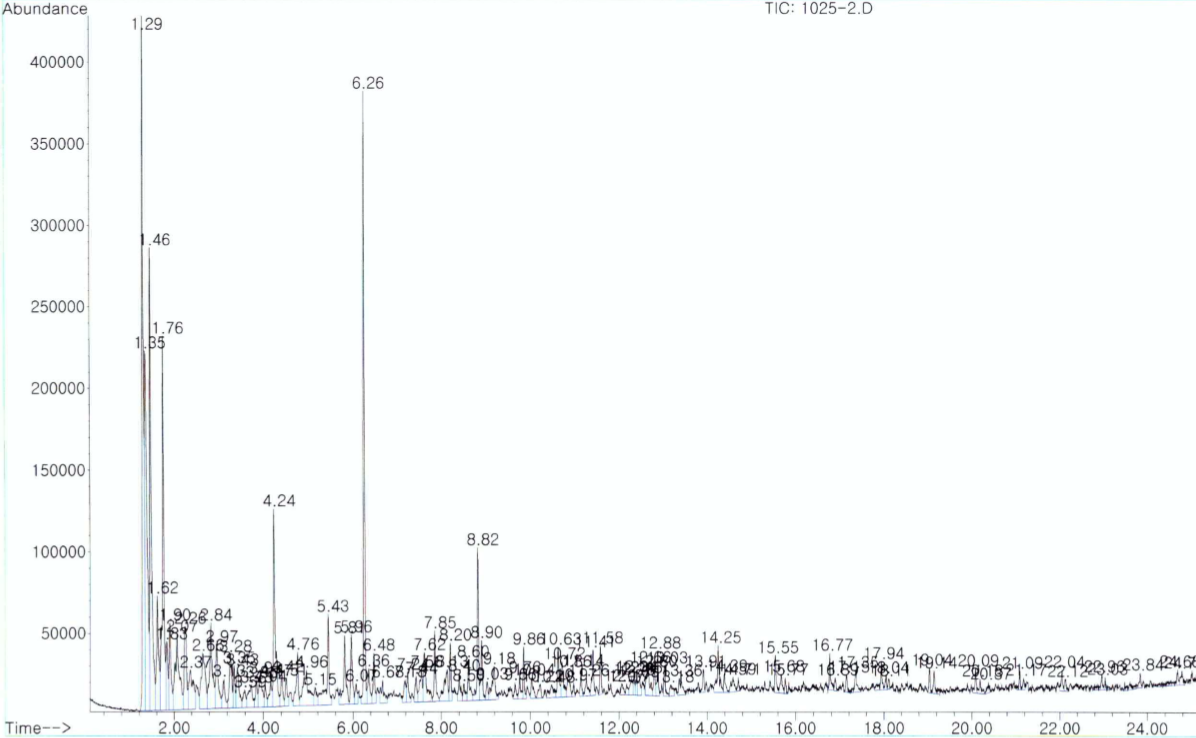 | S6  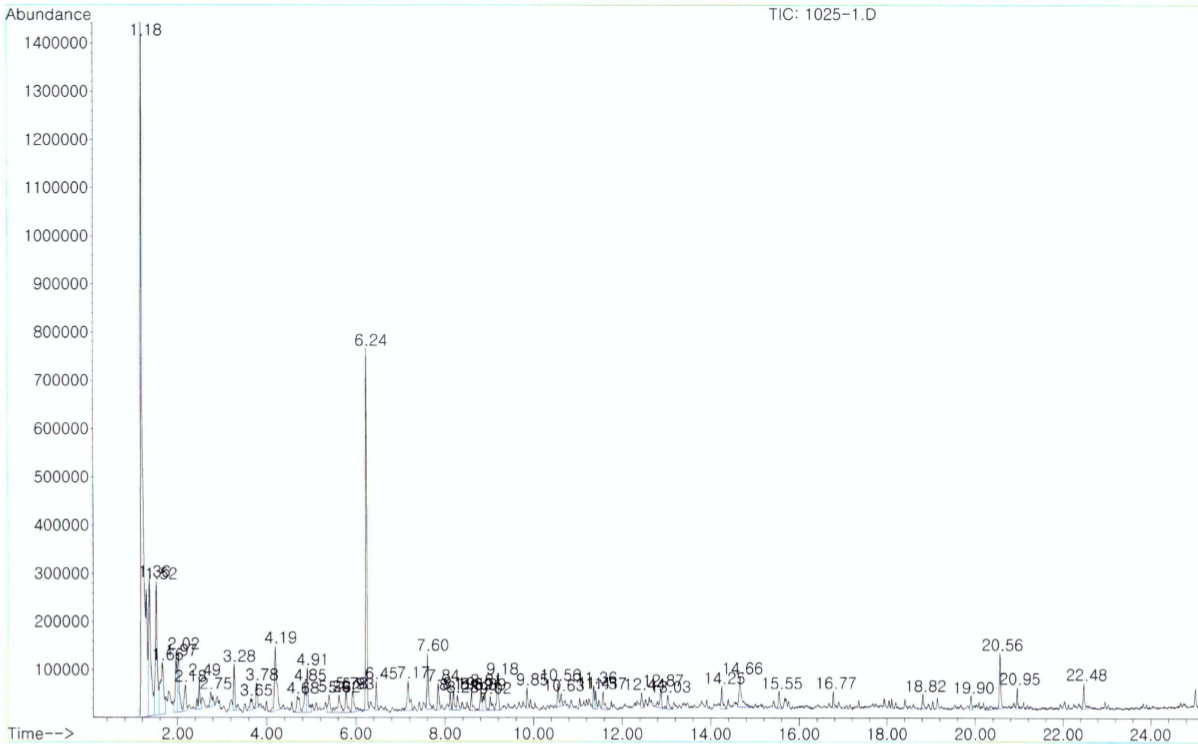 |
| S7  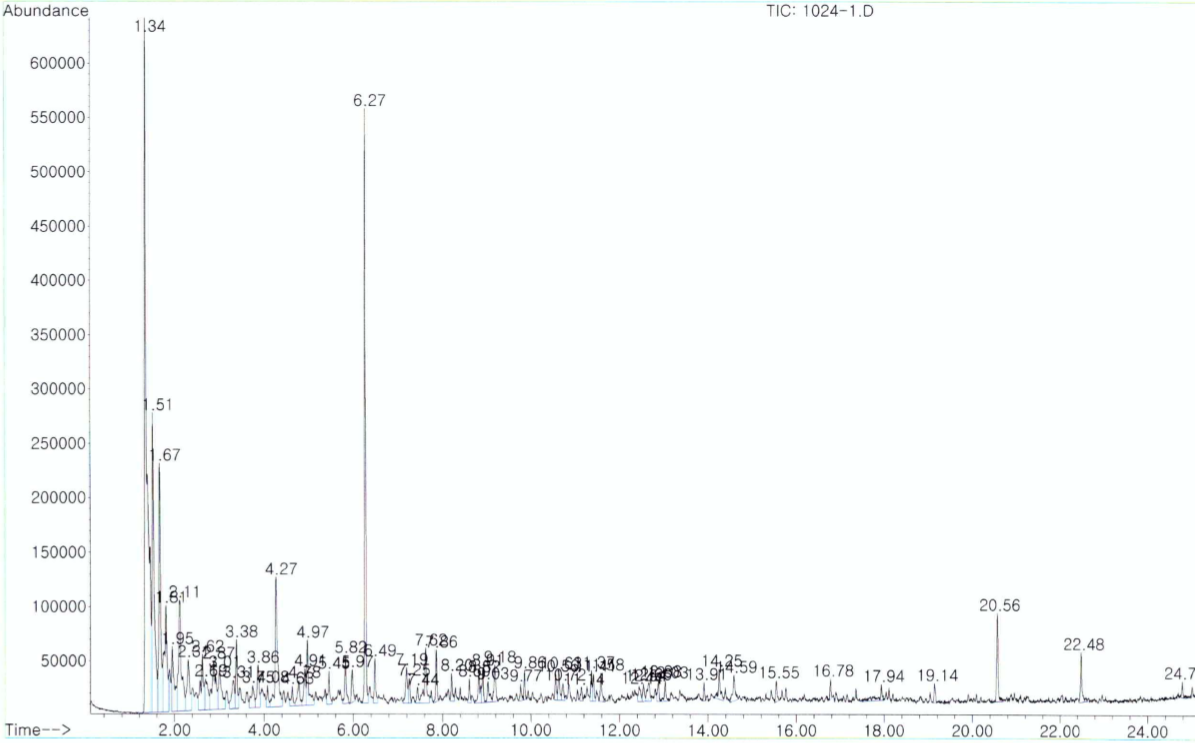 | S8  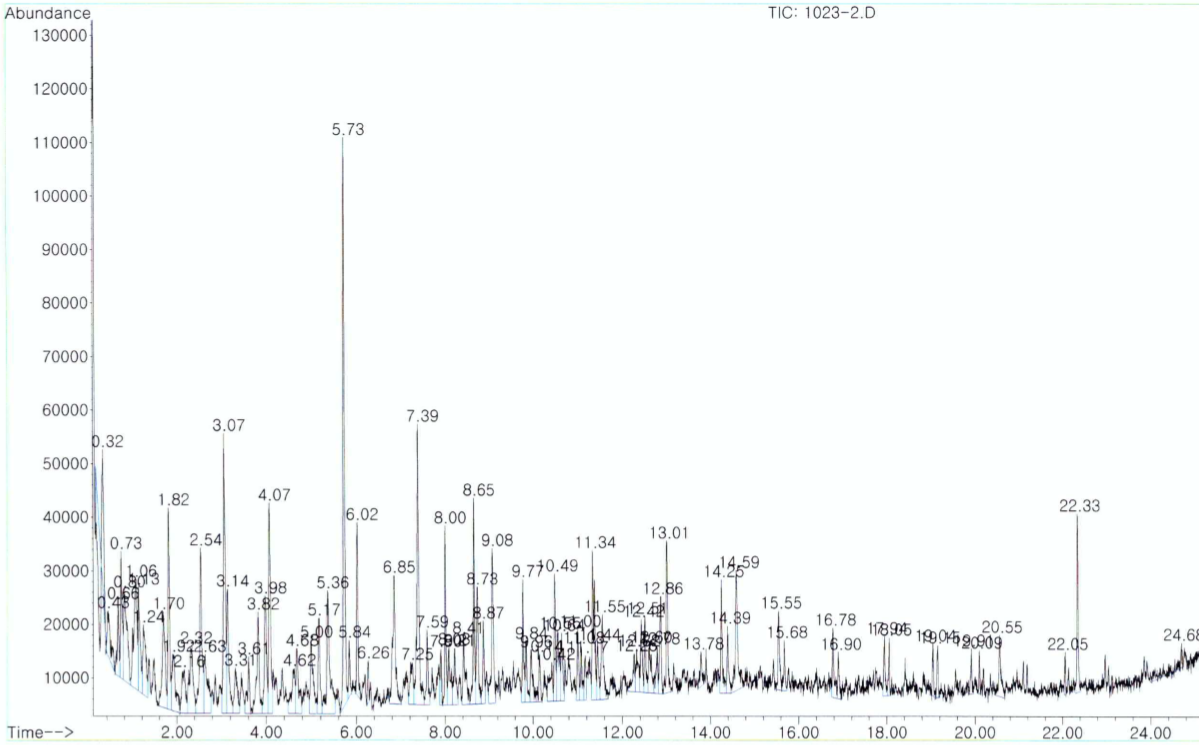 |
| S9  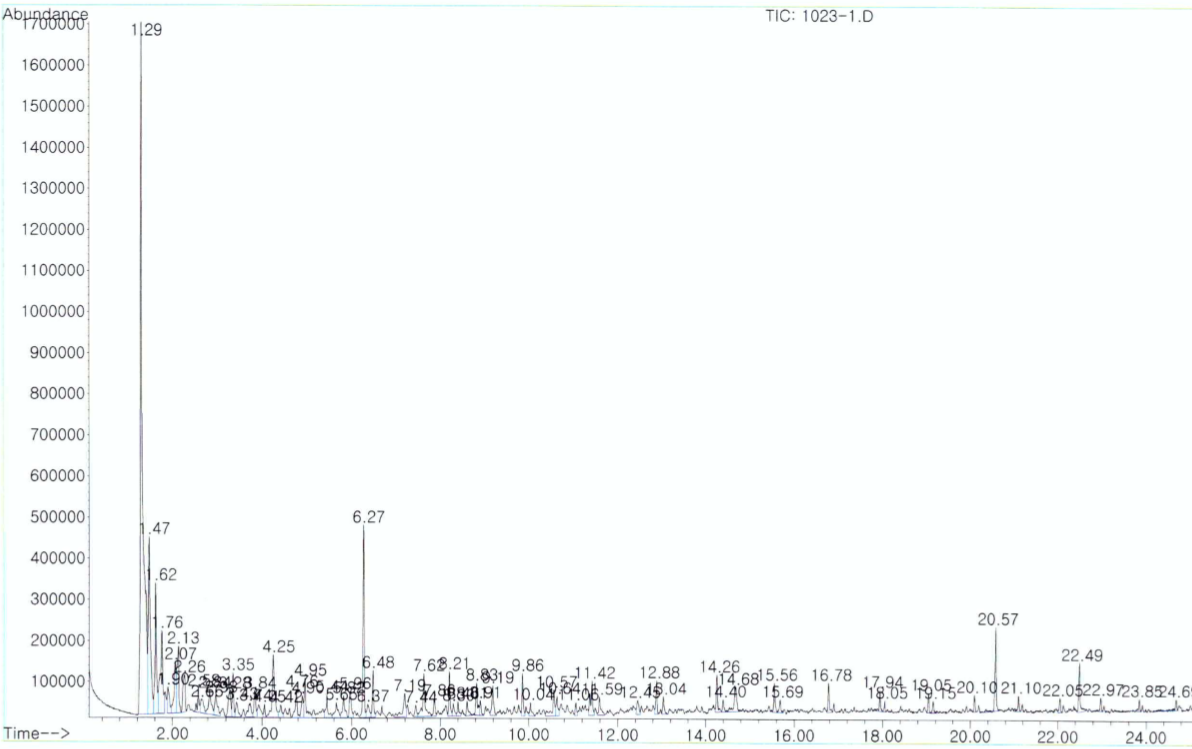 | S10  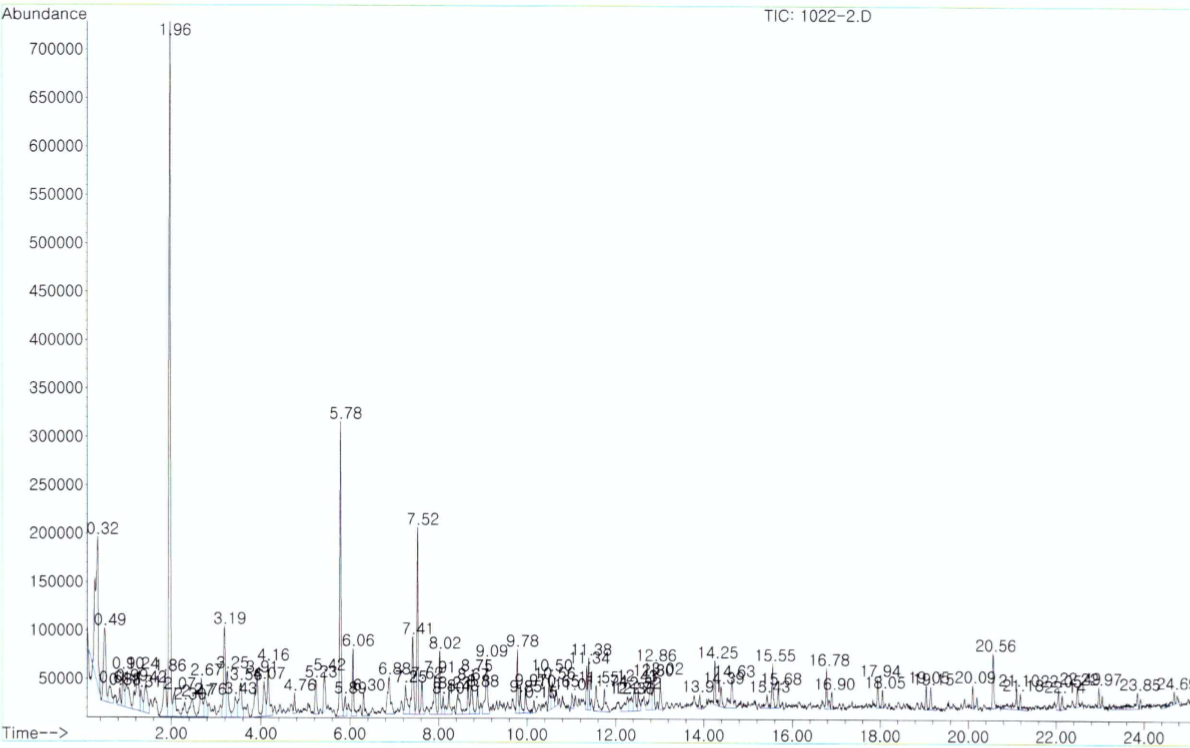 |
| S11  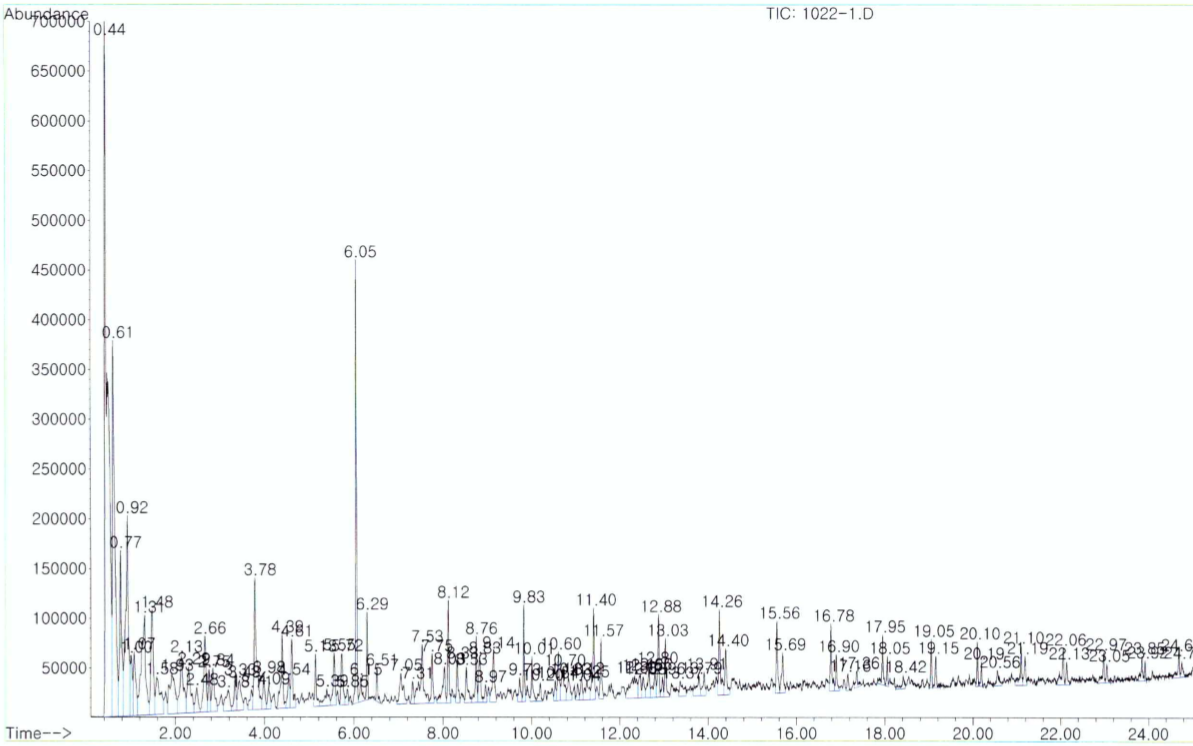 | S12  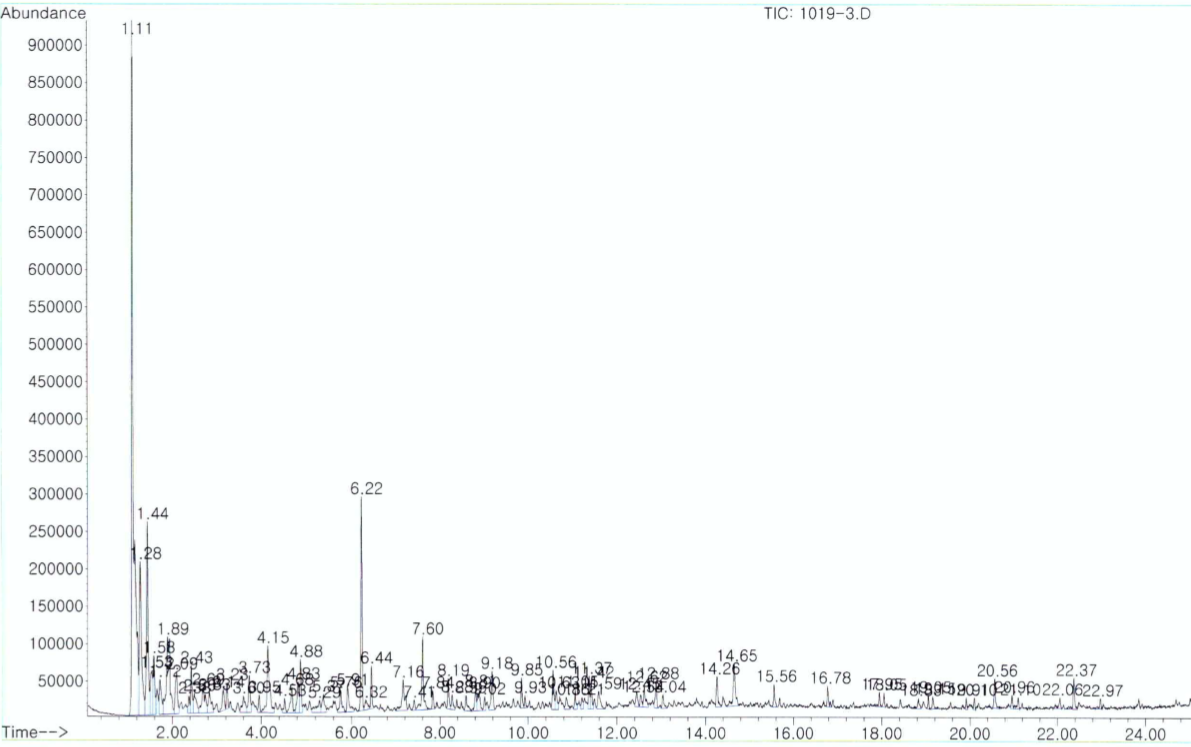 |
| S13  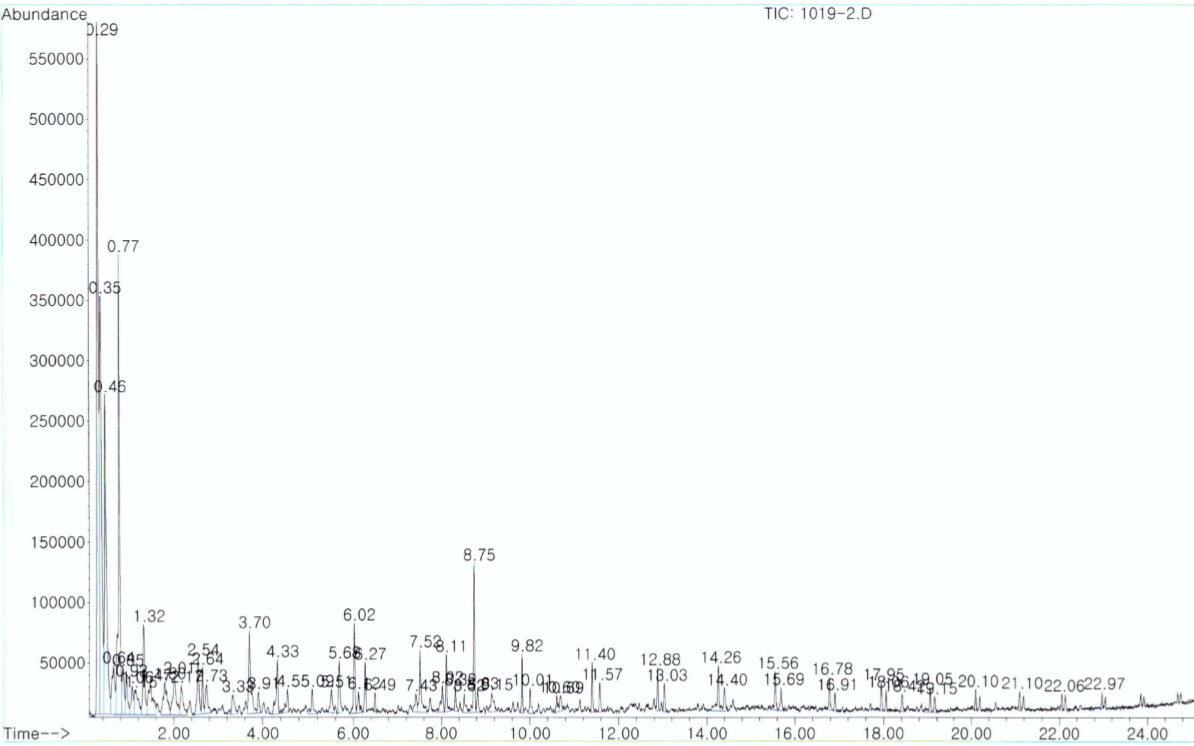 | S14  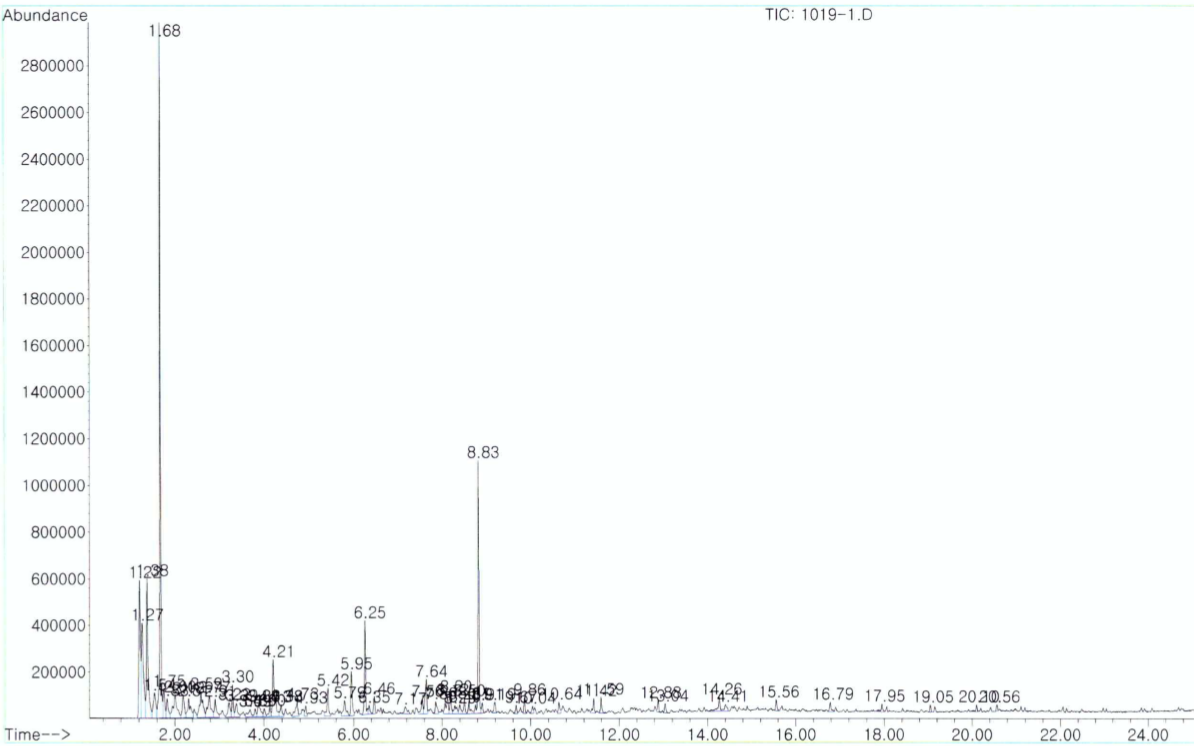 |
| S15  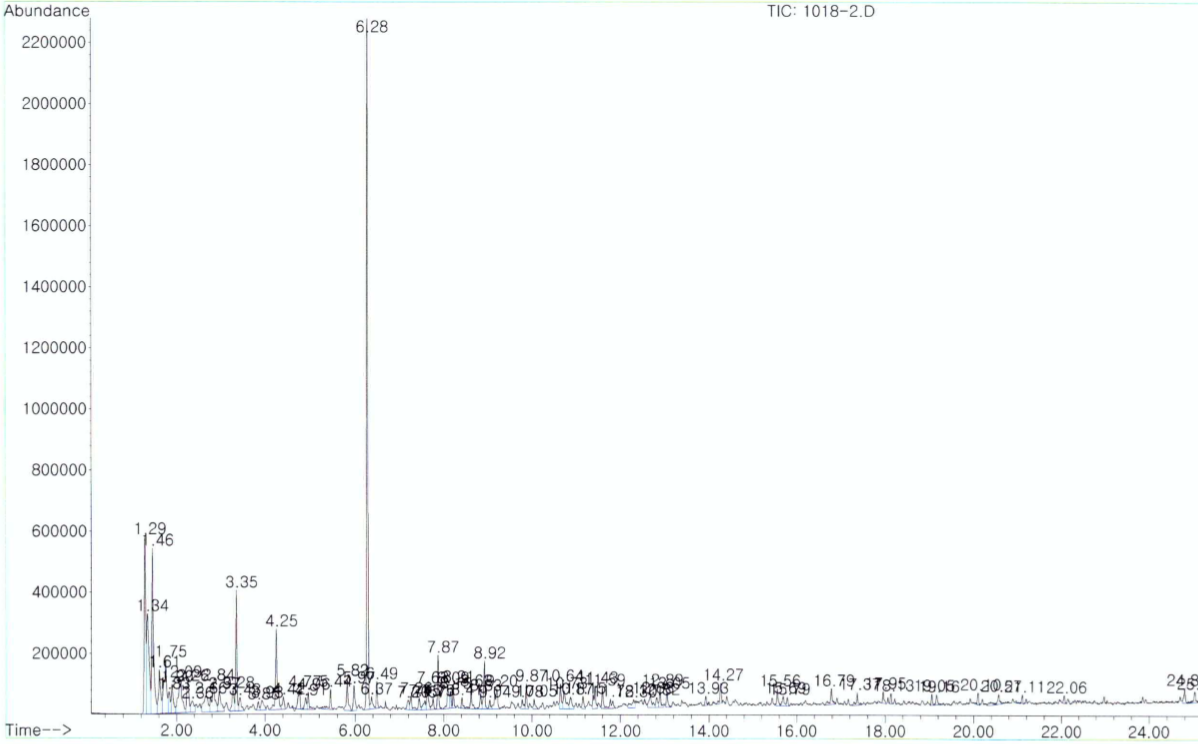 | S16  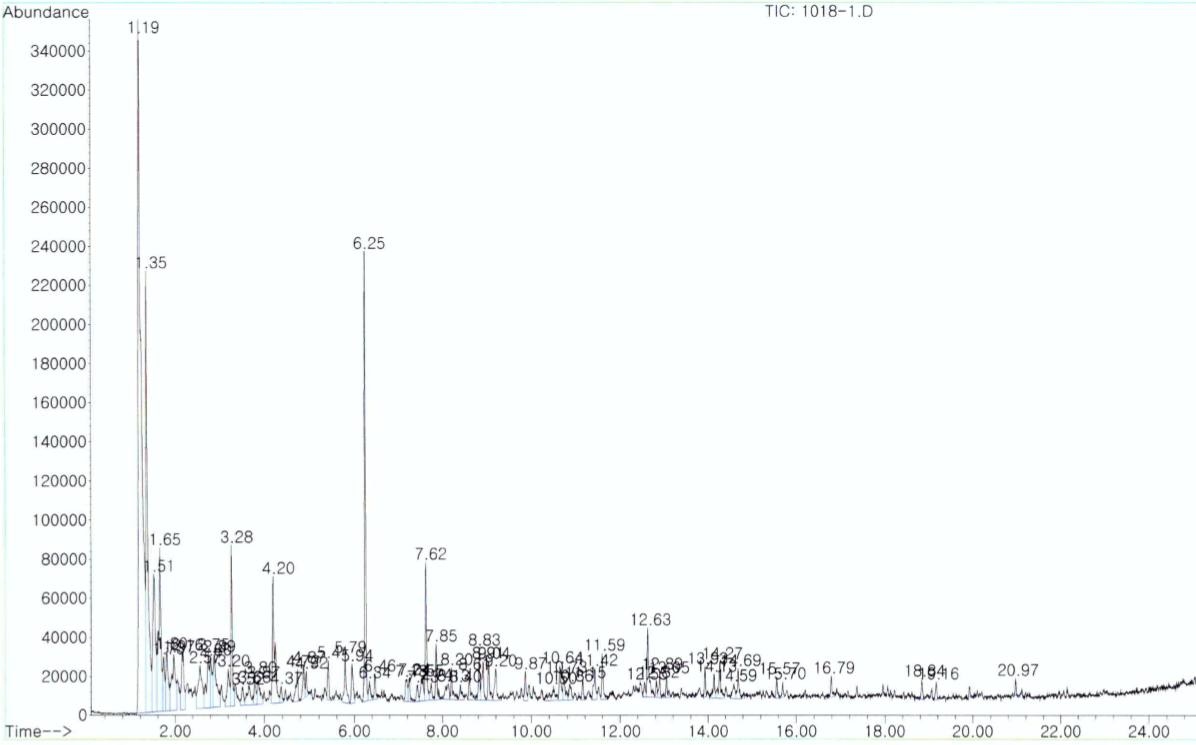 |
| S17  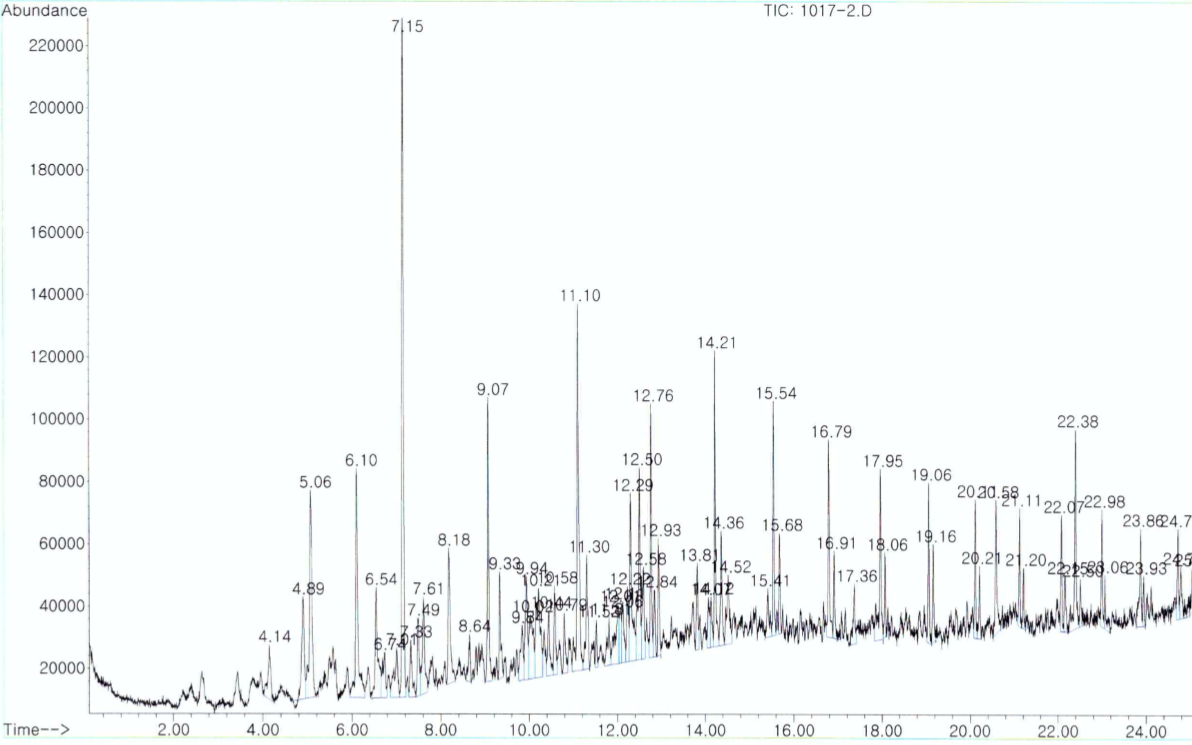 | S18  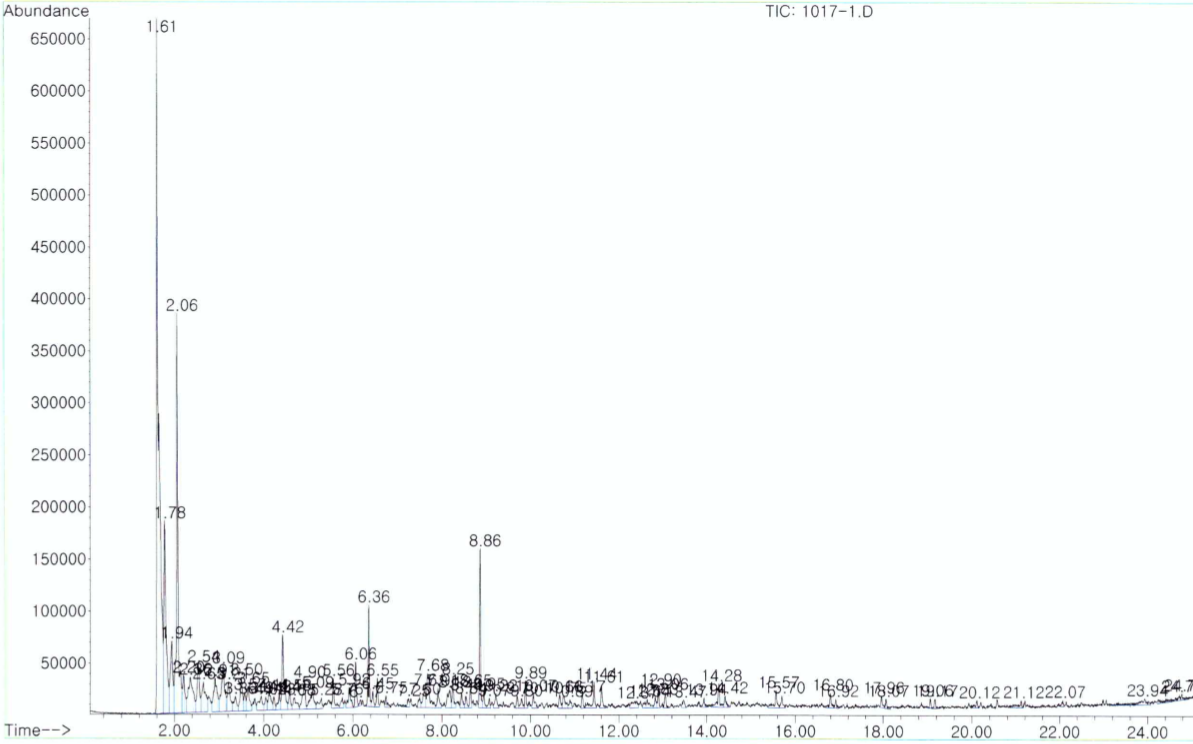 |
| S19  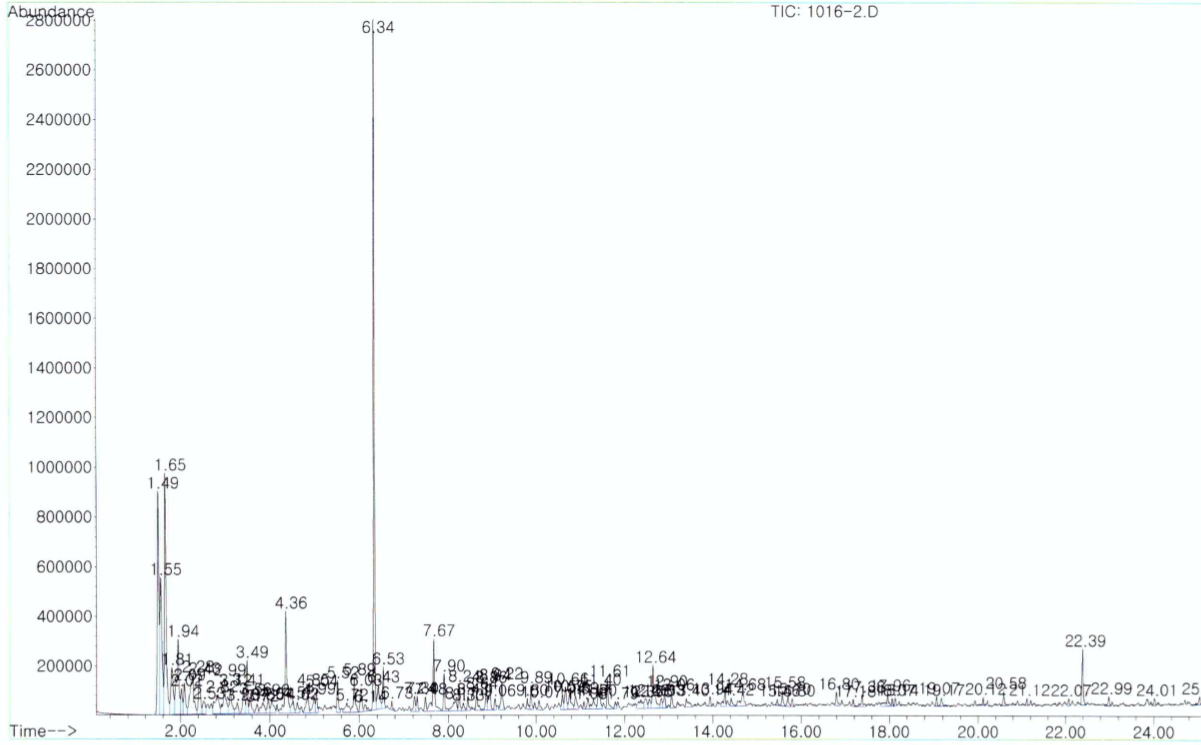 | S20  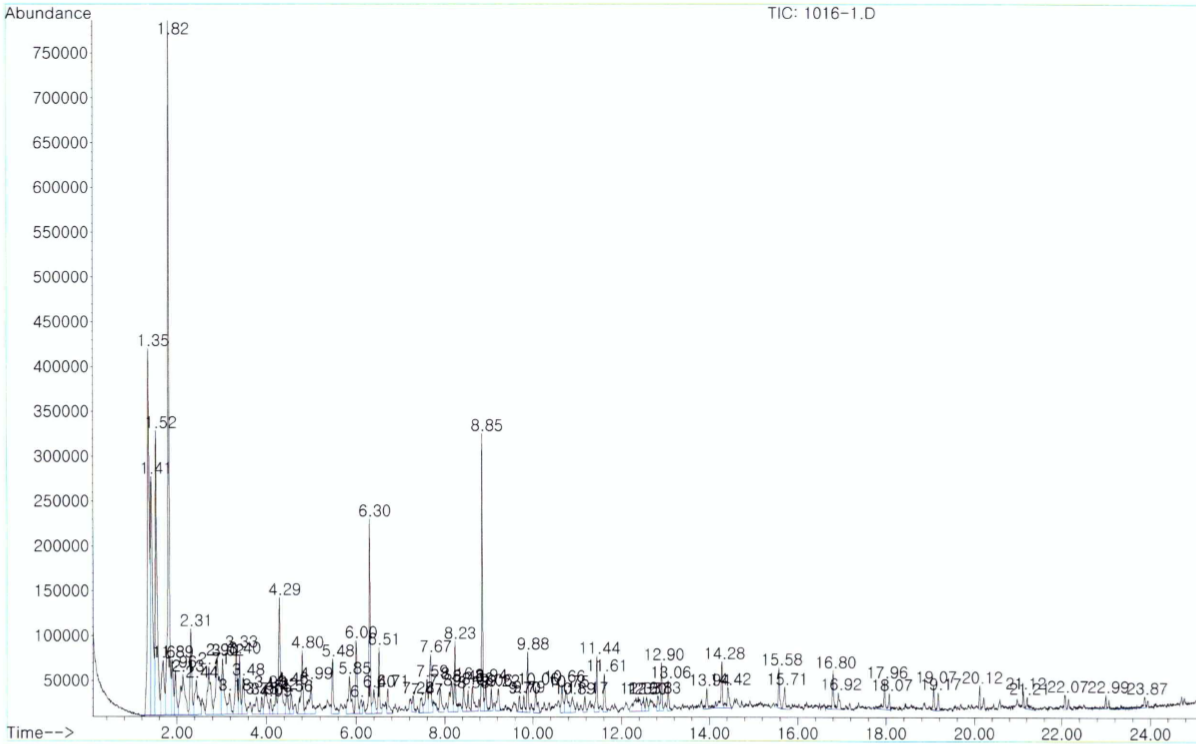 |
| S21  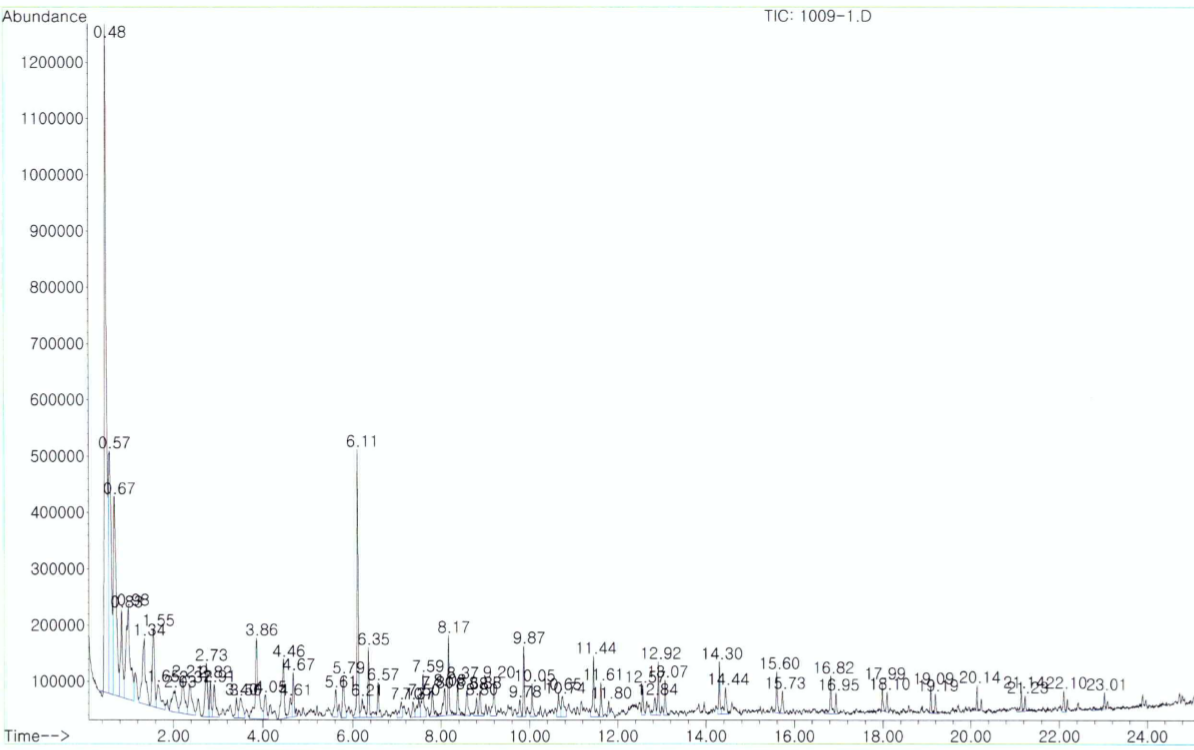 | S22  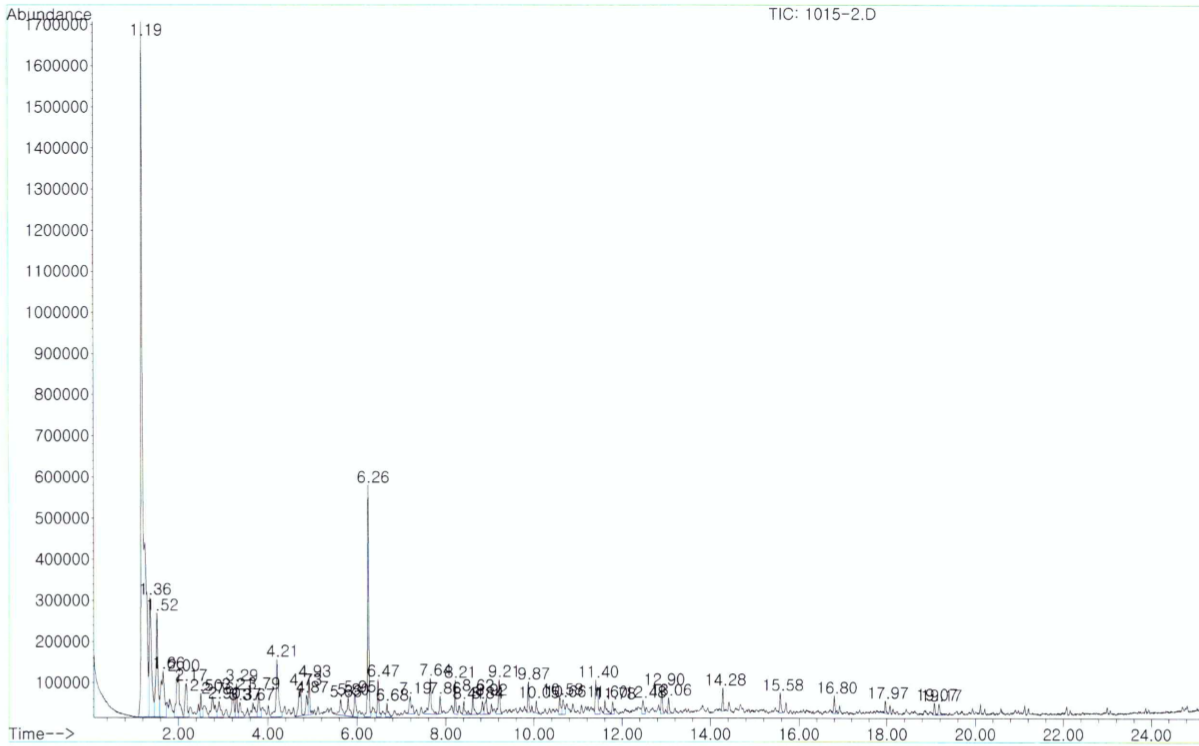 |
| S23  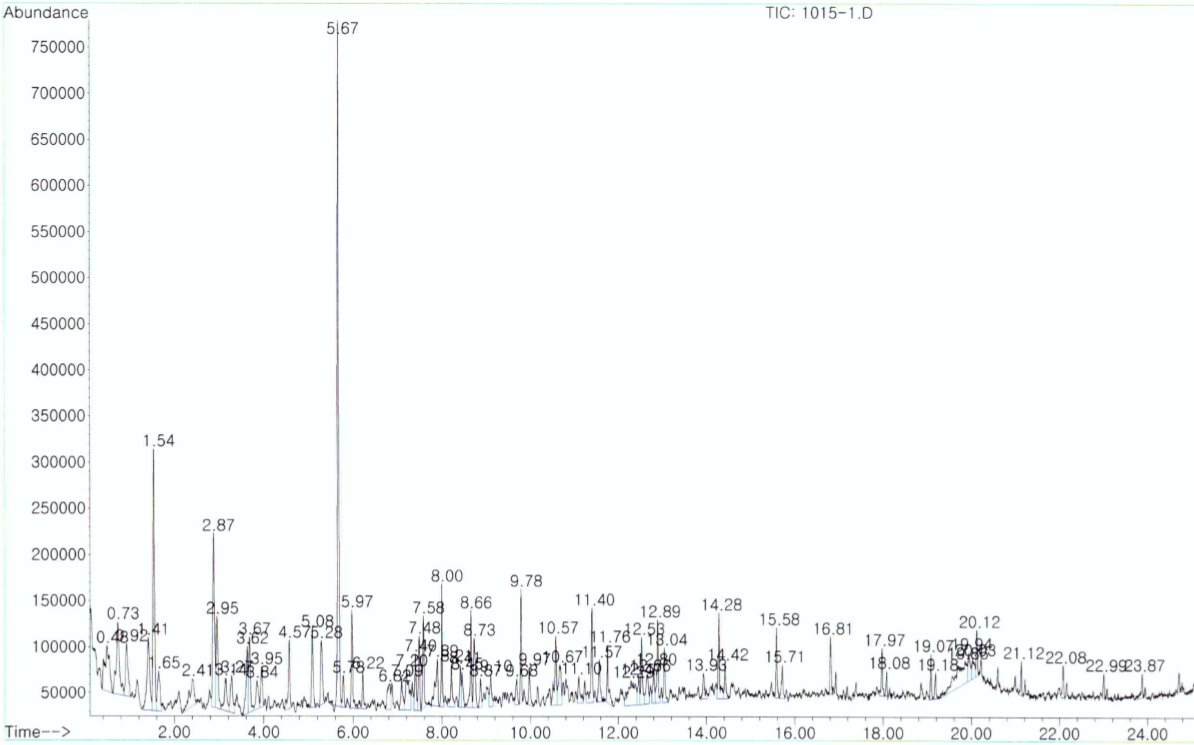 | S24  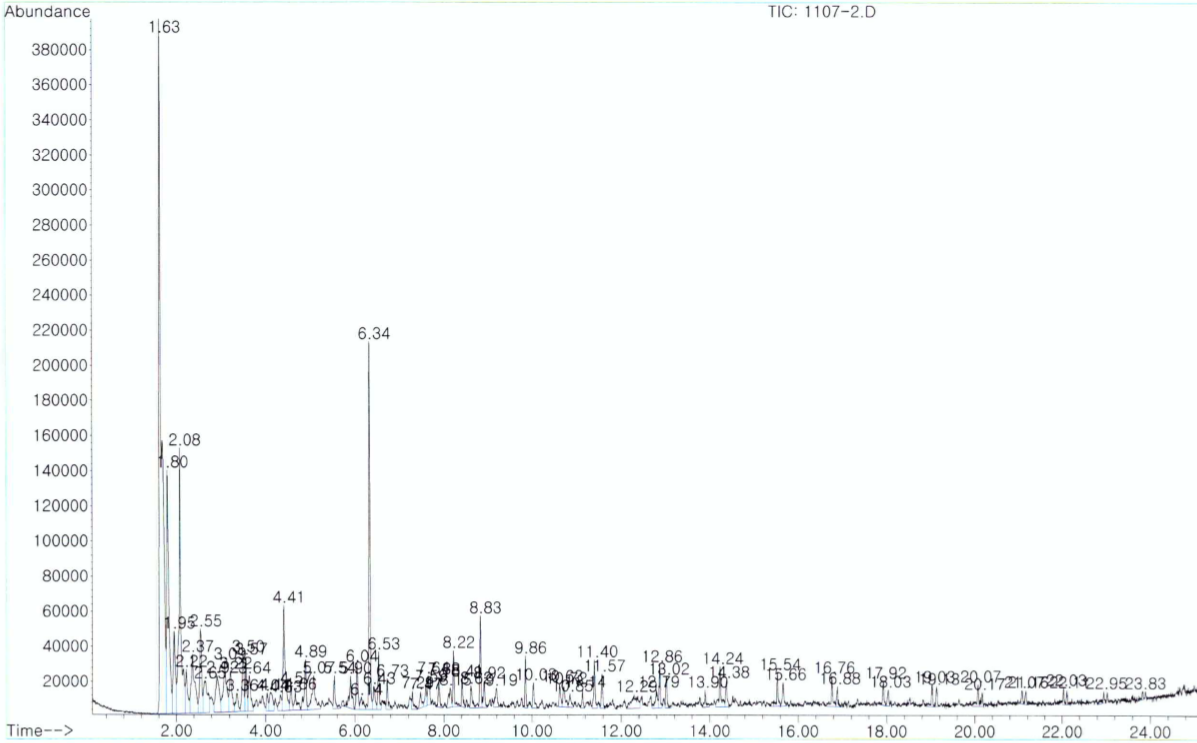 |
| S25  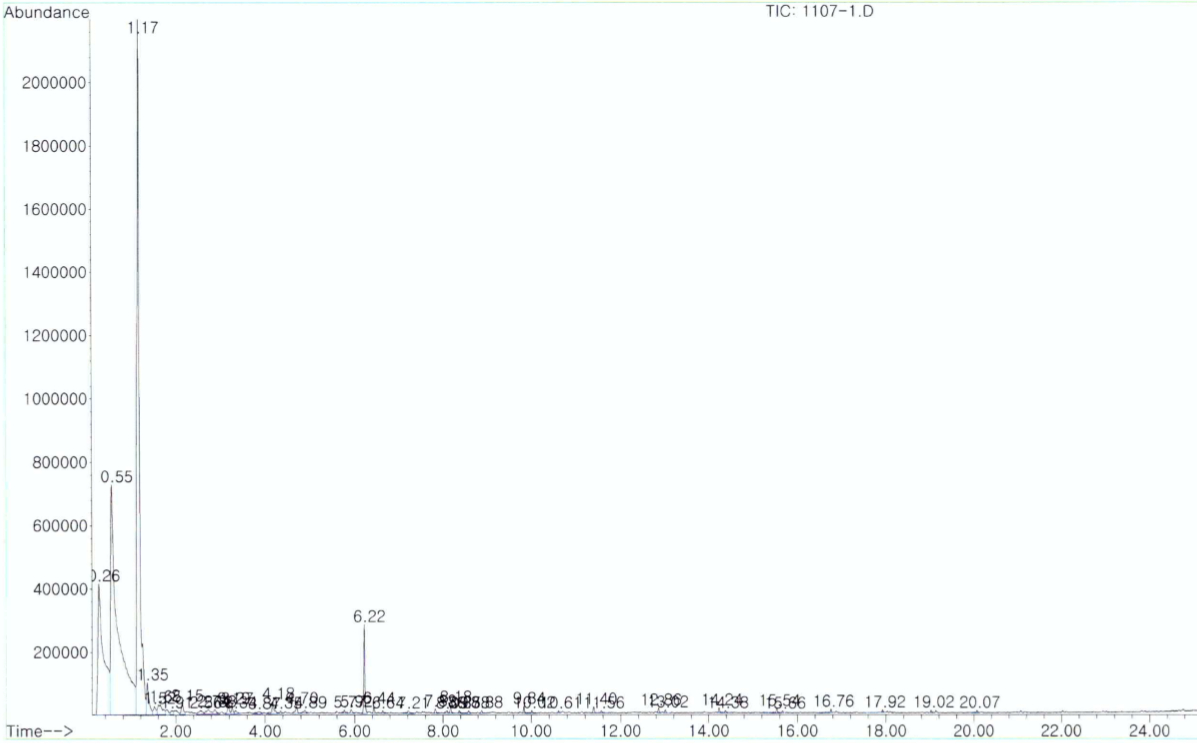 | S26  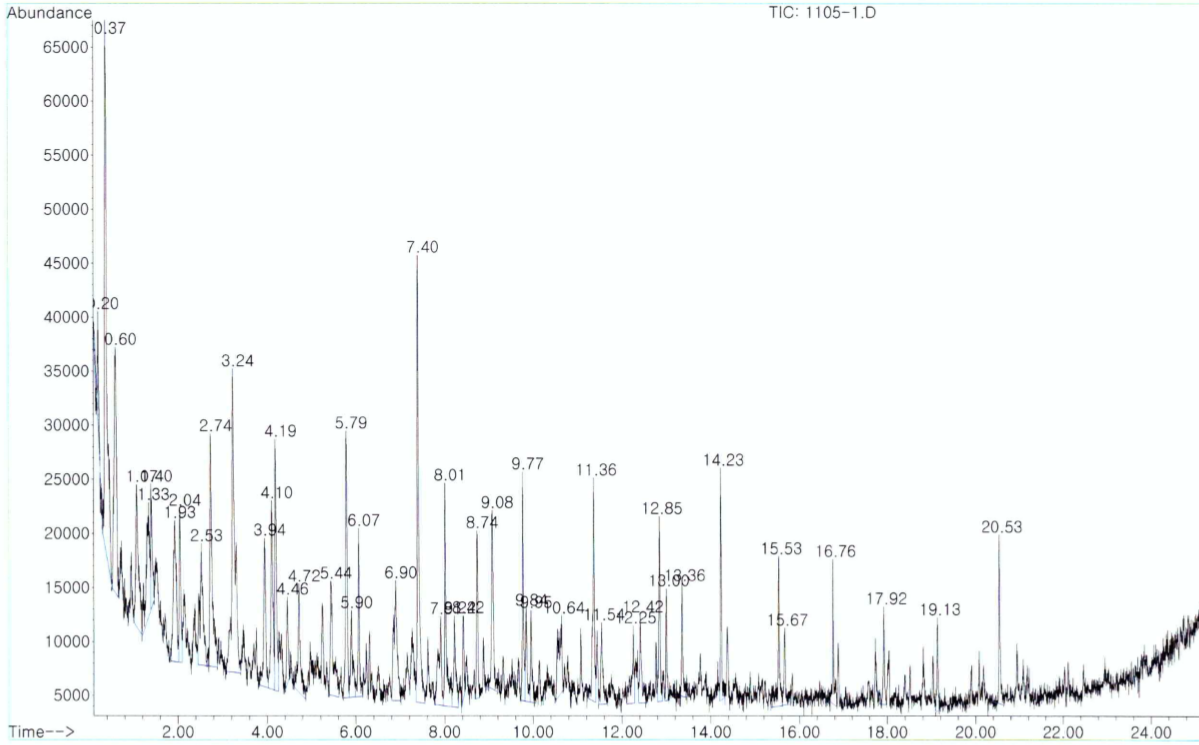 |
| S27  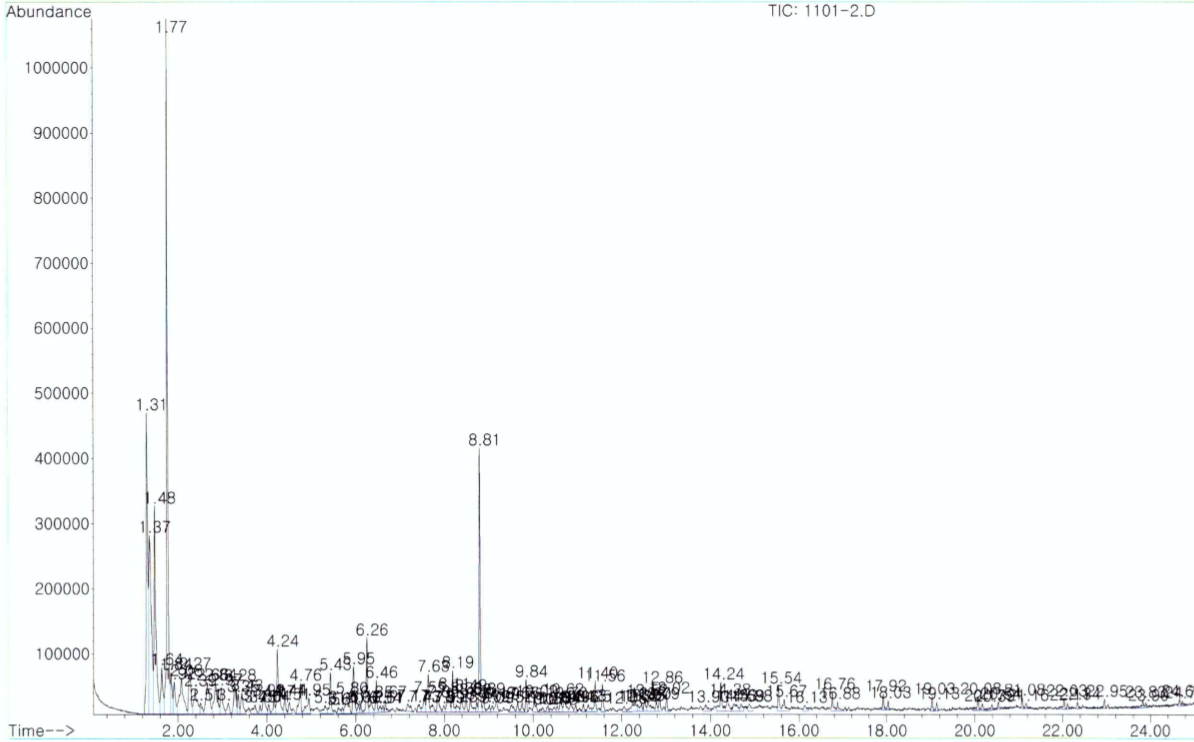 | S28  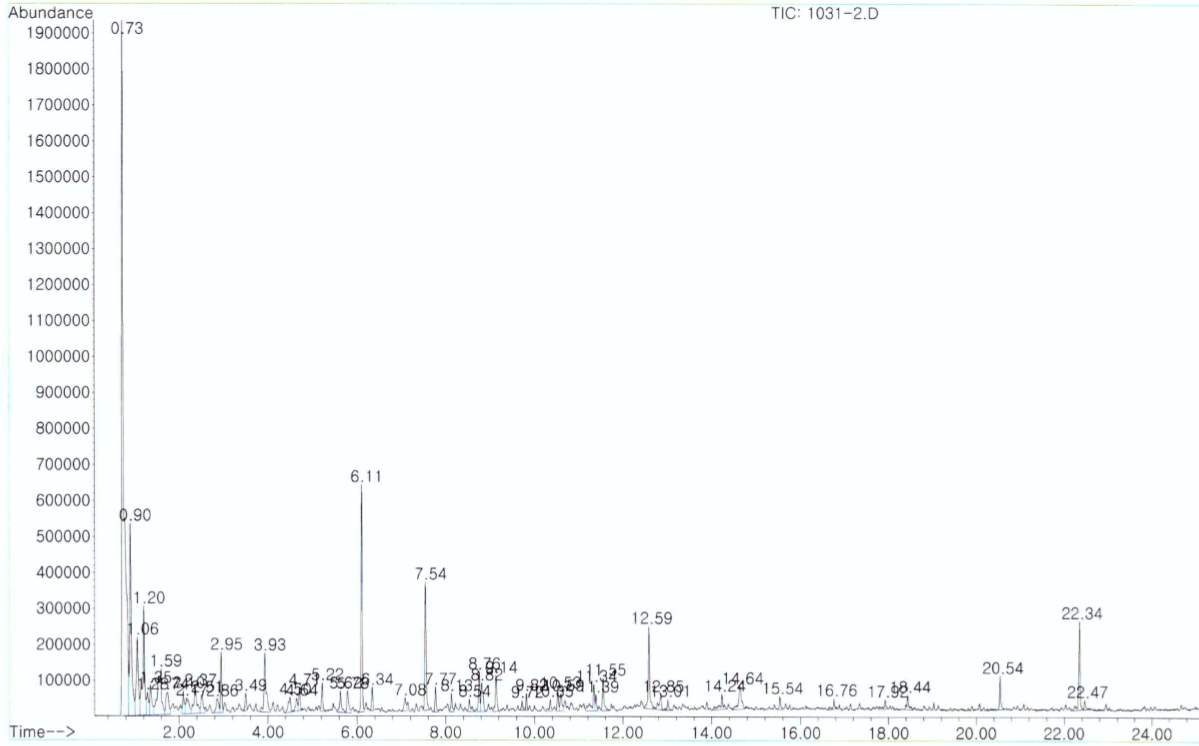 |

**Figure S3.** Py-GC-MS spectrum of each solid road dust sample from site S1-S28

**
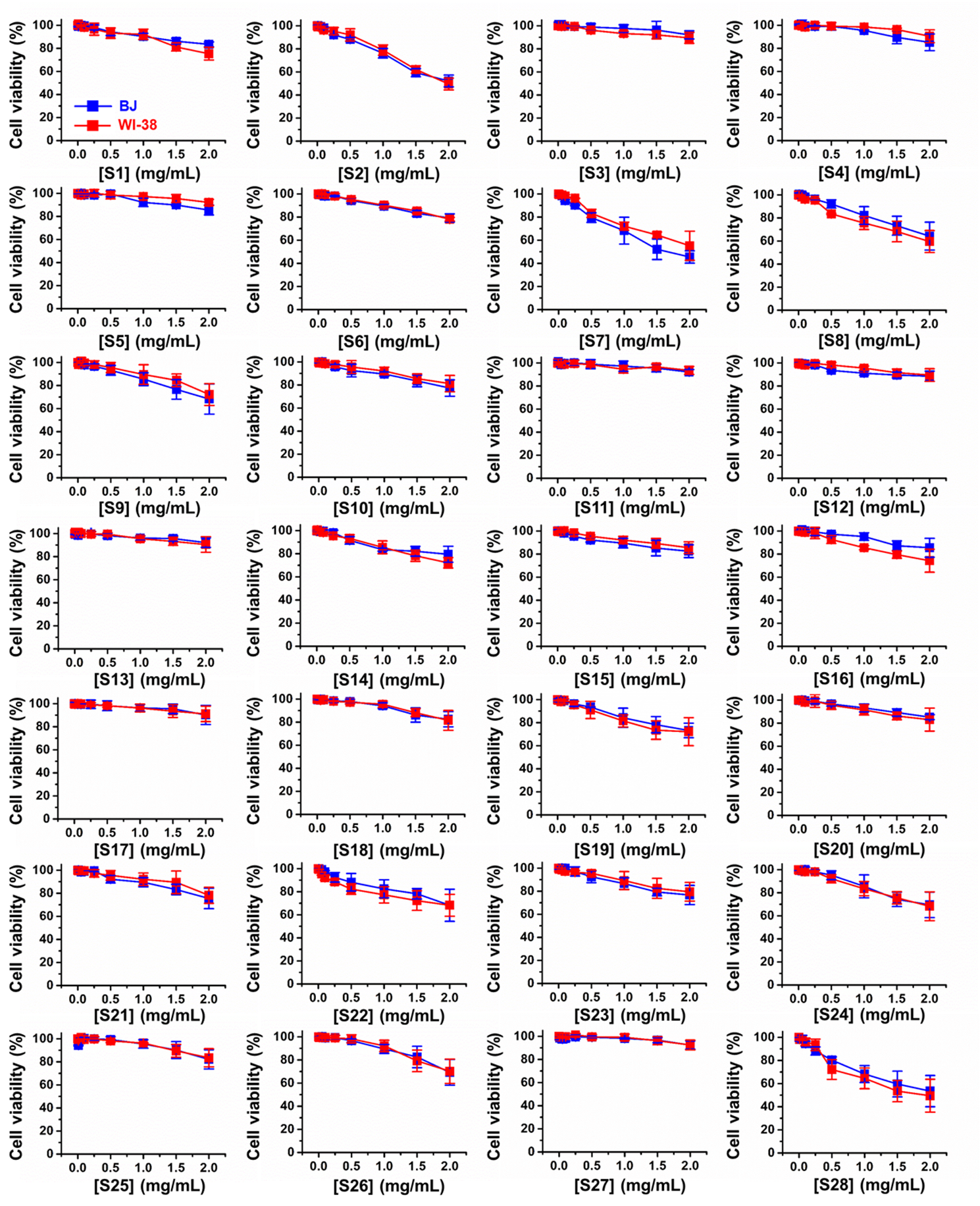
**

**Figure S4.** Dose-response relationship between urban road dust samples collected from 28 different areas and lung (WI-38) and skin (BJ) fibroblasts viability. The error bars correspond to standard deviations among the triplicate samples.


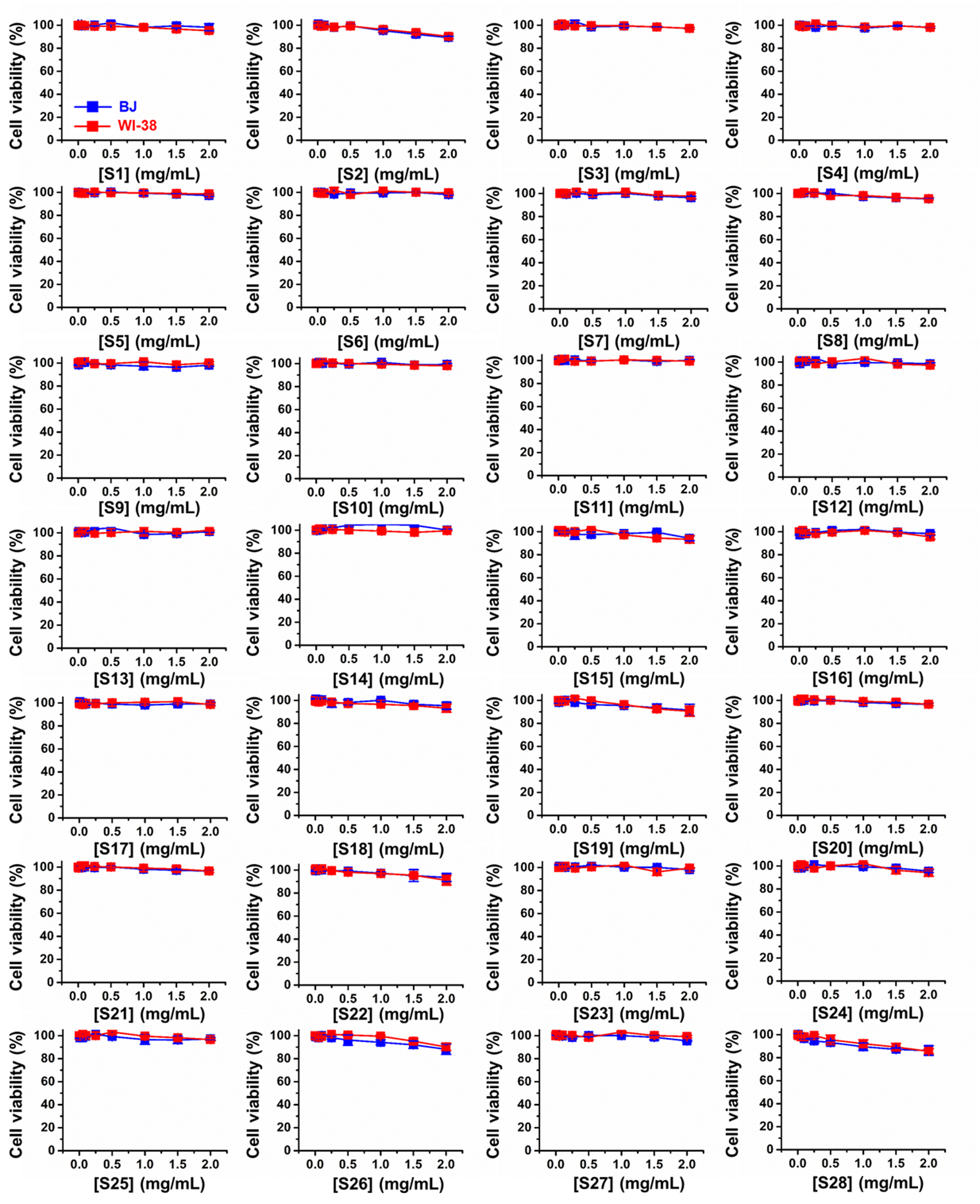


**Figure S5.** Dose-response relationship of water-extract part of urban road dusts collected from 28 different area and WI-38, BJ fibroblasts viability. The error bars correspond to standard deviations among the triplicate sample


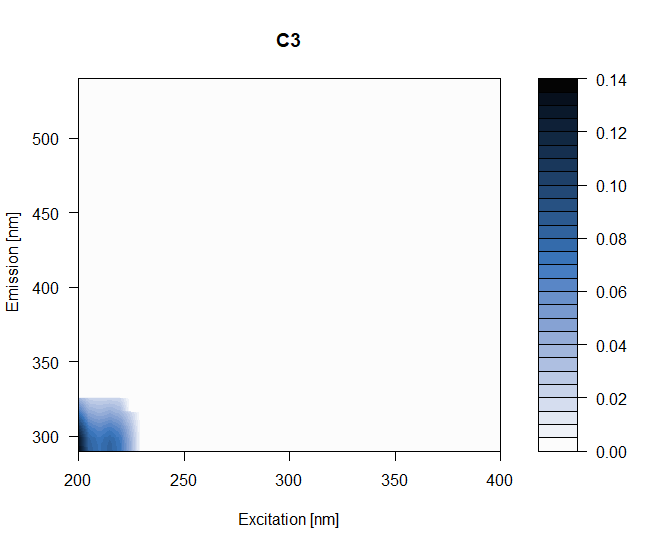


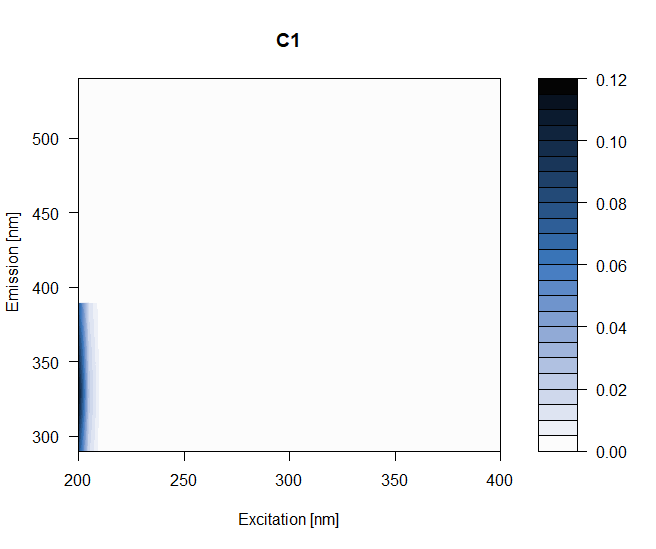

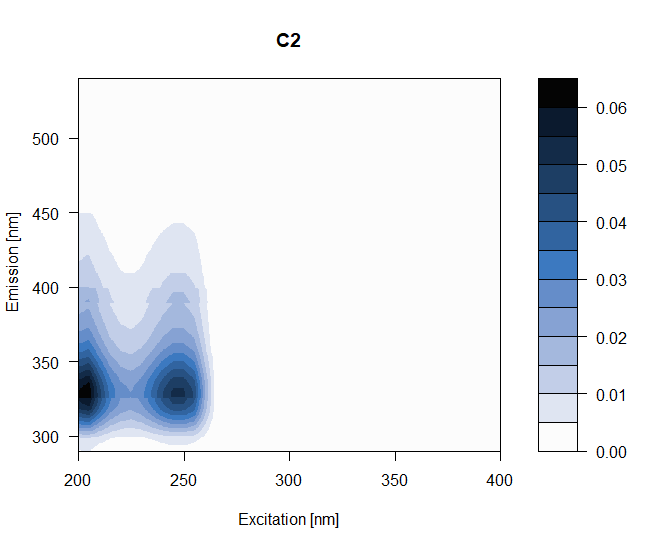


**Figure S6.** The three PARAFAC components that consist 112 EEMs obtained from water-extract of ultra-fine road dust
